# Wheat native endophytic bacteria isolated from Mediterranean and Atlantic agroecosystems: identification, functional profiling and seedling bioassays

**DOI:** 10.3389/fmicb.2026.1913855

**Published:** 2026-07-30

**Authors:** Adriano B. Bingobingo, Sandra Hilário, Nuno Mariz-Ponte, Natasha Ortolan, Tânia R. Fernandes, Susana M. P. Carvalho

**Affiliations:** 1 GreenUPorto—Sustainable Agrifood Production Research Centre/Inov4Agro, DGAOT, Faculty of Sciences of the University of Porto, Vila do Conde, Portugal; 2 Department of Rural Engineering, Faculty of Agricultural Sciences of José Eduardo dos Santos University, Chianga, Huambo, Angola

**Keywords:** bioinoculants, biostimulants, climate change, nutrient use efficiency, plant growth promoting bacteria, salinity stress, water deficit

## Abstract

Wheat (*Triticum aestivum*) cultivation is increasingly challenged by climate change, scarcity of natural resources and dependence on synthetic fertilizers. Plant growth-promoting bacteria (PGPB) represent a promising, sustainable approach to increase crop resilience and enhance resource-use efficiency. However, most studies have primarily focused on PGPB isolated from specific geographical regions and predominantly from the rhizosphere, thereby limiting the exploration of microbial diversity that may harbor novel strains with distinct functional traits and enhanced biostimulant potential. Thus, this study explored wheat-native endophytic bacteria isolated across different agroecosystems in the Mediterranean and Atlantic region, integrating taxonomy, broad multi-trait assessment for functional profiling and seedling bioassays to explore PGPBs with potential for improvement of wheat resilience. A total of 181 isolates belonged to the families *Pseudomonadaceae, Bacillaceae* and *Paenibacillaceae*, from which 66 representatives were selected for downstream analysis. Most isolates exhibited relevant plant growth-promoting traits, including indole compounds production (95.5%), 1-aminocyclopropane-1-carboxylate (ACC) deaminase activity (100%), siderophore production (100%), and phosphate solubilization (62%). In addition, many isolates demonstrated tolerance to salinity and PEG-induced osmotic stress. Interestingly, substantial variability was observed among strains in the magnitude of these functional traits. Several isolates significantly enhanced wheat germination and early seedling growth, with *Pseudomonas tritici* (B254) increasing total biomass by 35.2%. In contrast, *Bacillus pumilus* (B393) and *Pseudomonas* sp. (B451) showed inhibitory and suppressive effects on germination and seedling development. These findings reveal a wide range of functional diversity among wheat endophytic bacteria and highlight promising native strains for the development of bioinoculants to improve wheat performance.

## 1 Introduction

Global food security remains one of the major challenges of our time, due to the increasing population, rising consumer demand for sustainably produced food, climate change and resource limitations (United Nations Department of Economic and Social Affairs (Un Desa) 2021). Wheat (*Triticum aestivum*) is a staple crop of major importance for global food security, providing essential nutrients to approximately 2.5 billion people from countries where it serves as a primary dietary component (Gupta et al., 2021; CGIAR, 2022; Dangi et al., 2023). Beyond its nutritional value, wheat cultivation supports economies worldwide through cultivation, processing and marketing, and plays a central role in international trade, contributing to economic stability and strengthening connections between nations (Dangi et al., 2023; Arreyndip, 2025). The European Union (EU) is a key player in global wheat production. In 2025, the EU harvested over 143 million tons (+ 16.8% compared to 2024), and accounting for 18% of the world's wheat output (Eurostat, 2025; FAOSTAT, 2026). In 2024, the EU ranked as the second- largest wheat producer globally after China, with Ireland leading in production per hectare, followed by the United Kingdom and Denmark (FAOSTAT, 2026). Looking ahead, wheat production and exports in the EU are projected to increase 1.38 million tons (around 10%) by 2033 and potentially positioning the EU as the world's leading wheat producer (Oecd/Fao, 2024). However, achieving this goal will require overcoming rising environmental challenges such as water scarcity, soil degradation, salinization and heat waves (Tian et al., 2021). Moreover, wheat commercial cultivation heavily relies on synthetic fertilizers to guarantee high yields, but their intensive use has generated serious environmental and health concerns, including soil degradation, biodiversity loss, water contamination, and increased greenhouse gas emissions, ultimately threatening the sustainability of wheat production (Tian et al., 2023; Zhang et al., 2023; Elumalai et al., 2025). Consequently, there is an urgent need to develop and adopt sustainable agricultural strategies that maintain a high wheat productivity while minimizing the environmental impacts. Among these, plant growth-promoting microorganisms have emerged as promising alternatives to improve nutrient use efficiency (NUE) and enhance crop resilience under adverse environmental conditions. In addition to single-strain inoculants, microbial consortia have attracted increasing attention due to their ability to integrate complementary plant growth-promoting mechanisms and improve crop performance under abiotic stresses, including salinity (Gholizadeh et al., 2026; Kaya et al., 2026).

Beneficial microorganisms are increasingly emerging as relevant tools to face the aforementioned challenges. Since July 2022, microbial biostimulants have been formally recognized under the EU Fertilizing Products Regulation, reflecting their role in enhancing growth and NUE. Their importance was further emphasized at the 2024 United Nations Climate Change Conference (COP29), where microbial-based solutions were highlighted as key strategies to mitigate climate change impacts in agriculture. In this context, plant growth-promoting bacteria (PGPB), especially members of the pseudomonads and bacilli groups, including the genera *Pseudomonas, Bacillus* and *Paenibacillus*, have attracted considerable attention owing their multifunctional roles in enhancing plant growth and performance (Maciel-Rodríguez et al., 2025; Valente et al., 2025). They promote nutrient mobilization and availability, while modulating plant growth through phytohormones synthesis and mitigating stress through ACC deaminase activity (Alzate Zuluaga et al., 2024; Cheng et al., 2025; Cruz De Carvalho et al., 2025; Taheri et al., 2025).

Despite their potential, the effective adoption of PGPB in agriculture is often limited by variable efficacy under different soils, cropping systems and climates (Taheri et al., 2025). Moreover, most PGPB studies have focused on strains from specific regions and predominantly from rhizosphere habitats, limiting access to the broader microbial diversity that may harbor novel strains with enhanced biostimulant potential (Taheri et al., 2025). Sampling across contrasting agroecosystems offers a key strategy to capture this diversity and identify candidates with distinct adaptive traits. Accordingly, the present work forms part of a broader survey of the wheat microbiome across different agricultural practices and soil types, focusing here, through culture-dependent approaches, on endophytic bacteria from the families *Pseudomonadaceae, Bacillaceae*, and *Paenibacillaceae*, known for their biostimulant potential. Thus, the specific aims of this study are to: (i) isolate and phylogenetically identify wheat endophytes obtained from different agroecosystems in Mediterranean and Atlantic regions; (ii) perform a functional profiling of selected strains focusing on multi-trait assessment associated with tolerance to water deficit, salinity stress and enhanced NUE; and (iii) evaluate the effects of selected strains on wheat seed germination and early seedling development, providing insights into their potential as bioinoculants for sustainable and climate-resilient wheat production.

## 2 Material and methods

### 2.1 Sampling and bacterial isolation

Between June and July 2023, wheat samples (roots, leaves and grains) were collected from distinct geographical locations and agroecosystems to isolate a broad range of endophytic microorganisms with potential plant growth-promoting traits. This included one location in Spain (Zamora), which has a Mediterranean climate, and four locations in the Netherlands (Lelystad, Westmaas, Valthermond and Nieuw-Beerta), characterized by an Atlantic climate. In Spain, three fields with distinct agricultural practices (conventional irrigated, conventional non-irrigated, and organic non-irrigated) were selected, all under the same cultivar. In the Netherlands, fields representing four soil types (medium clay, heavy clay, peat-sand, and very heavy clay) and five wheat cultivars were sampled across both commercial and experimental plots managed by the Wageningen University & Research (Wageningen, NL). In both agroecosystems, within each field, nine biological replicates (n = 9) were collected per compartment, each consisting of a composite sample of five plants.

Sampling was carried out during the milky growth stage (BBCH 70–79) and to ensure minimal disturbance of the roots, plants were carefully uprooted and gently shaken to remove the excess of attached soil. Using a sterilized scissor, the root-shoot intersection was cut to 1–2 cm from the roots, and these were placed in sterile plastic bags (20 × 28 cm). Healthy leaves (no visible disease symptoms) were selected and collected in a sterile plastic bag, while ears were cut from the spikes and placed in 50 mL Falcon tubes, respectively. Upon sampling, all samples collected (*n* = 189) (Supplementary Table S1), were placed on dry ice and immediately transported to GreenUPorto—Faculty of Sciences of University of Porto. All samples were sterilized before bacterial isolation. In brief, plant tissues were surface disinfected with 70% (v/v) ethanol (1 min) and 5% (v/v) sodium hydrochloride (2 min) following three times washing with sterile deionized water (SDW). Around 20 mg of fresh matter from each tissue per 1 mL of SDW was used for isolation. The effectiveness of surface sterilization was confirmed by plating the final rinse water onto LB agar. No microbial growth was observed after incubation, indicating successful removal of epiphytic microorganisms. Each preparation was cultured on Luria Bertani (LB) agar medium and allowed to grow at 25 °C for 3–7 days. Single colonies were carefully chosen and streaked four to five times on new LB agar plates to obtain pure bacterial colonies. Bacterial stocks were prepared with 25% (v/v) glycerol and stored at – 80 °C. A serial dilution of the tissue extracts (10?^1^ to 10?^3^) was performed and 100 μL were spread on the top of each plate. Pure cultures are kept in the UPORTO microbial stock collection.

### 2.2 DNA extraction and PCR amplification

Genomic DNA was extracted from 24 h old cultures grown in LB medium and using the Bacterium DNA (BSC96S1E) extraction kit (Bioer Technology, China), according to the manufacture's protocols and using the MGISP-NE32 automated nucleic acid extraction platform (MGI Biotechnology, China). The quality and purity of the isolated DNA were determined using a μDrop™ plate (Thermo Fisher Scientific™, USA) read on the Multiskan SkyHigh Microplate Spectrophotometer (Thermo Fisher Scientific™, USA). For the molecular typing of all isolates, a BOX-PCR fingerprinting technique was carried out as described by (Versalovic, 1994). PCR reactions were performed in a volume of 25 μL, containing: 15.75 μL of sterile water, 6.25 μL of NZYTaq 2 × green Master Mix (Nzytech^TM^, Portugal), 2 μL of BOXA1R primer (Table 1) (STABVida, Portugal) at 10 pmol/μL and 1 μL of DNA template (≈ 400 ng μL ^−1^). PCR reactions were conducted in a Bio-Rad C1000 Touch thermal cycler (USA) following the conditions described by (Versalovic, 1994). After amplification, 1.5% agarose gels were stained with GreenSafe Premium (Nzytech, Lisbon, Portugal) and visualized under UV light (ChemiDoc Imaging System, Bio-Rad, USA).

**Table 1:** Primers used in this study.

Loci	Primer	Sequence 5^′^-3^′^	Target family	References
**–**	BOXA1R	CTACGGCAAGGCGACGCTGACG	All	Versalovic (1994)
16 rRNA	27F	AGRGTTTGATCMTGGCTCAG	All	Lane (1991)
	1492R	GGTCCTTGTTACGACTT		
*gyrB*	*gyrB-Fps*	MGGCGGYAAGTTCGATGACAAYTC	*Pseudomonadaceae*	Sarkar and Guttman (2004)
	*gyrB-Rps*	TRATBKCAGTCARACCTTCRCGSGC		
	*gyrB-Fbs*	CGTAGAGGGWGAYTCTGC	*Bacillaceae and Paenibacillaceae*	Unpublished work
	*gyrB-Rbs*	CCAAGACCTTTRTAACGYTG		
*rpoD*	*rpoD-Fp*	AAGGCGARATCGAAATCGCCAAGCG	*Pseudomonadaceae*	Sarkar and Guttman (2004)
	*rpoD-Rps*	GGAACWKGCGCAGGAAGTCGGCACG		

Analysis of the fingerprinting patterns was performed with GelJ software (Heras et al., 2015). The Pearson correlation coefficient was applied, and cluster analysis was performed using the unweight pair group method with arithmetic mean (UPGMA) algorithm. The resulting dendrograms were analyzed to obtain groups of isolates with at least 85% similarity. This cutoff was determined so that patterns that were known to be equal (molecular weight size markers) would be in the same group (Fidalgo et al., 2016). The 16S rRNA, *gyrB* and *rpoD* genes were selected as molecular barcodes for bacterial identification. Amplification of 16S rRNA was performed using the universal primers 27F/1492R (Table 1), and PCR conditions were as followed as described by (Lane, 1991). Part of the *gyrase B* gene (*gyrB*) was also amplified for all isolates (Table 1) following the conditions described by Sarkar and Guttman (2004), respectively. Part of the *rpoD* gene was amplified for the family *Pseudomonadaceae* only, using the primer set rpoD-F/rpoD-R (Table 1) and following the PCR conditions as described by Sarkar and Guttman (2004). All PCR reactions were prepared in a final volume of 25 μL using NZYTaq 2 × Green Master Mix, as described above. PCR reactions were conducted using a Bio-Rad C1000 Touch thermal cycler (USA). Amplicons were purified with the NZYGelPure Kit (Nzytech™, Portugal) and sequenced by STABVida (Lisbon, Portugal).

### 2.3 Identification and phylogenetic analysis

The nucleotide sequences were analyzed with FinchTV v.1.4.0 (Geospiza, Inc. Seattle, Washington, USA; www.geospiza.com/finchtv). The 16S rRNA sequences obtained were compared against the Standard Nucleotide BLAST (https://blast.ncbi.nlm.nih.gov/) for identification at the genus level. Although the initial 16S rRNA BLAST screening revealed the presence of isolates tentatively assigned to other bacterial families, only those belonging to *Pseudomonadaceae, Bacillaceae*, and *Paenibacillaceae* were retained for in-depth species-level identification and functional characterization. This decision was based on three criteria: (i) their numerical dominance within the collection, collectively accounting for the majority of recovered isolates (57.1%); (ii) their well-documented plant growth-promoting potential and biotechnological relevance in cereal crops; and (iii) the scope of the present study, which was designed to focus on these taxa within the broader WHEATBIOME project framework. Isolates assigned to other families were deposited in the UPORTO microbial stock collection for future investigation. Clade assignments were initially assessed through an initial 16S rRNA phylogenetic tree containing all validly published species of *Bacillus, Peribacillus, Paenibacillus, Terribacillus, Solibacillus, Psychrobacillus, Pseudomonas* and *Phytopseudomonas* according to the List of Prokaryotic names with Standing in Nomenclature (LPSN, August 2025) (https://lpsn.dsmz.de/). To refine species-level assignments, multilocus sequence analysis was performed using housekeeping genes: 16S rRNA and *gyrB* for *Bacillus, Peribacillus, Paenibacillus, Terribacillus, Solibacillus* and *Psychrobacillus*, and 16S rRNA, *gyrB* and *rpoD* for *Pseudomonas* and *Phytopseudomonas*. Annotated genomes of closely related representative strains from the NCBI database were used to retrieve their corresponding 16S rRNA, *gyrB*, and *rpoD* sequences (Supplementary Table S2). Sequences were aligned using ClustalX v.2.1 software (Larkin et al., 2007) with pairwise and multiple alignment set as default. Alignments were optimized and manually edited using BioEdit Alignment Editor v.7.0.5. (Hall, 1999) and concatenated using Sequence Matrix (Vaidya et al., 2011). Maximum likelihood (ML) analyses were conducted in MEGA v.11.0 (Tamura et al., 2021) starting from a Neighbor-Joining tree automatically generated by the software. Nearest Neighbor Interchange (NNI) was used as the heuristic method for tree inference and 1,000 bootstrap replicates were performed. MEGA v.11.0 was also used to determine the best nucleotide substitution model for constructing the ML trees. Phylogenetic trees were edited in Inkscape Vector software v.1.1 (https://www.inkscape.org) (Adobe Systems Inc, San Jose, CA, USA). Sequences generated in this study were deposited in GenBank (Supplementary Table S3).

### 2.4 Functional profiling of the selected strains

Representative bacterial strains identified through BOX-PCR fingerprinting within the *Pseudomonadaceae, Bacillaceae*, and *Paenibacillaceae* families were subsequently subjected to functional profiling using a multi-trait assessment approach. To that end, ten biochemical assays were performed to evaluate plant growth-promoting traits grouped into three categories: (i) plant performance and stress resilience (Indole-Acetic Acid-equivalents—IAA; 1-aminocyclopropane-1-carboxylate—ACC Deaminase; pH; salt and osmotic stress tolerance) (Gupta and Pandey, 2019b; Orozco-Mosqueda et al., 2020); (ii) nutrient acquisition and NUE improvement (nitrogen fixation; urease; phosphate solubilizing index; siderophores production) (Rawat et al., 2021; Jeong et al., 2024); and (iii) colonization ability (bacterial motility) (Liu et al., 2024b). It should be noted that not all biochemical assays were applied to the full set of 66 representative isolates. A first-tier screening comprising IAA-equivalents production, phosphate solubilization, and nitrogen fixation was conducted on all 66 strains to enable an initial ranking and selection of the most promising candidates (Supplementary Table S4). Based on this screening, a subset of 32 isolates, comprising the nine top-performing *Pseudomonas* strains and all 23 members of the *Bacillaceae* and *Paenibacillaceae* families, was subsequently subjected to second-tier assays, including ACC deaminase activity, siderophore production, urease activity, motility, and tolerance to pH, salinity, and osmotic stress. For biochemical assays, uninoculated media were used as negative controls to confirm that the detected responses were associated with bacterial activity. For stress tolerance assays, growth under non-stress conditions was used as the reference control.

#### 2.4.1 Indole-acetic acid-equivalents (IAA)

The indole compounds production was assessed as described previously by Ferreira et al. (2021), with slight modifications. Bacteria were grown in LB medium at 28°C for 24 h. Fresh single colonies, adjusted to an OD_600_ of 0.5, were inoculated on 5 mL of LB medium amended with 1% (w/v) L-tryptophan (Thermo Fisher Scientific™, USA) and incubated in an orbital shaker for 24 h at 28 °C and 180 rpm. Salkwoski solution (2 mL 0.5 M FeCl_3_ in 98 mL of 35% (v/v) perchloric acid) was added to the cell free culture supernatant (2:1) and incubated in the dark for 30 min. Development of red color (which indicates the formation of indolic compounds) was measured spectrophotometrically at 530 nm (Multiskan SkyHigh ThermoScientific™, USA). The concentration of IAA was determined with a standard curve of commercial IAA (Thermo Fisher Scientific™, USA) ranging between 0 and 100 μg mL^−1^, and the results are reported as IAA-equivalent concentrations, since the Salkowski assay is not specific for IAA.

#### 2.4.2 ACC deaminase

A colorimetric method, using cultures grown in liquid Dworkin and Foster (DF) medium supplemented with 3 mM ACC (Thermo Fisher Scientific™, USA), was used for the quantitative analyses of ACC deaminase activity, expressed as the amount of α-ketobutyrate produced. Isolates grew in liquid DF minimal salt medium, containing KH_2_PO_4_ (4 g L^−1^), Na_2_HPO_4_ (6 g L^−1^), MgSO_4_·7H_2_O (0.2 g L^−1^), glucose (2 g L^−1^), gluconic acid (2.0 g L^−1^), citric acid (2.0 g L^−1^), FeSO_4_·7H_2_O (1 mg L^−1^), H_3_BO_3_ (10 μg L^−1^), MnSO_4_ (10 μg L^−1^), ZnSO_4_ (70 μg L^−1^), CuSO_4_ (50 μg L^−1^), MoO_3_ (10 μg L^−1^), with pH 7.2 (Ferreira et al. 2021). The OD_540_ of a sample was compared with that of a standard curve constructed with 0.1 to 1.0 μmol mL^−1^ solutions of alpha-ketobutyrate (Merck, Germany) (Penrose and Glick, 2003). DF medium without ACC was used as a negative control, while DF supplemented with 2.0 g L^−1^ of (NH4)_2_SO4 served as the positive control.

#### 2.4.3 Presumptive nitrogen-fixation

For the detection of nitrogen-fixation ability, isolates, previously cultured in LB medium and adjusted to an OD_600_ of 0.5, were inoculated in a semi-solid and free-nitrogen medium composed of glucose (10 g L^−1^), malate (10 g L^−1^), K_2_HPO_4_ (1 g L^−1^), CaCl_2_ (0.1 g L^−1^), MgSO_4_·7H_2_O (0.2 g L^−1^), FeSO_4_·7H_2_O (0.01 g L^−1^), Na_2_MoO_4_·2H_2_O (0.01 g L^−1^), MnSO_4_·5H_2_O (0.01 g L^−1^), NaCl (25 g L^−1^), agar (5 g L^−1^) and 2 mL bromothymol blue (Yu et al., 2017). Nitrogen fixation was presumptively detected by the change of color of the medium from green to blue. Non-inoculated medium served as a negative control. Each isolate was tested in triplicate.

#### 2.4.4 Urease activity

Urease activity was assessed using a qualitative method based on the Christensen's urea agar medium (Pei et al., 2024), with slight modifications. The medium contained yeast extract (0.1 g L^−1^), KH_2_PO4 (0.091 g L^−1^), NaHPO4 (0.095 g L^−1^), phenol red (0.01 g L^−1^), agar (15 g L^−1^), and urea (20 g L^−1^), adjusted to pH 6.7 ± 0.2 at 25 °C. Five milliliters of the medium were dispensed into tubes, which were inclined during cooling until solidification. Pure bacterial colonies were inoculated in triplicate using a loop and incubated at 28 °C for 72 h. A color change from orange to pink in the medium indicated a positive urease reaction.

#### 2.4.5 Phosphate solubilizing index (PSI)

Phosphate solubilization was screened using the Pikovskaya (PVK) agar (HiMedia, Germany) method, adapted by Gupta and Pandey (2019a) and with minor modifications. The isolates were previously cultured in LB medium at 28°C for 24 h and adjusted to an OD_600_ of 0.5. Each bacterial isolate was aseptically inoculated (5 μL) at the center of a 90 mm Petri dish containing 15 mL of PVK medium. The plates were incubated for 7 d at 28 °C and visually monitored for the presence of halos around the colonies. The development of a clear halo around the bacterial colonies was considered as positive phosphate solubilizers, indicating the dissolution of tricalcium phosphate present in the medium. The diameter of the halos was measured using the ImageJ software (Schneider et al., 2012), and the values were recorded in mm. The phosphate solubilizing index (PSI) was calculated by using the following Equation [1]:


$$\begin{array}{l} PSI=\;\frac{Colony\;diameter+Halozone\;diameter}{Colony\;diameter} \end{array}$$ (1)

#### 2.4.6 Siderophore production

The siderophore production was determined by the Chrome Azurol S (CAS) assay following the microplate protocol described by Arora and Verma (2017). Bacterial strains were grown in LB medium at 28°C for 24 h. After incubation, cultures were centrifuged at 10,000 × g for 5 min, and the supernatants were collected for siderophore quantification.

Chrome Azurol S (CAS) reagent was prepared according to (Schwyn and Neilands, 1987) . Briefly, 121 mg of CAS (Merck, Germany) was dissolved in 200 mL of distilled water, added to 20 mL of 1 mM ferric chloride (FeCl3·6H_2_O) solution prepared in 10 mM HCl. This solution was then added to 20 mL of hexadecyl trimethyl ammonium bromide (HDTMA) solution (1.83 g L?^1^) freshly prepared and the pH was adjusted to 5.6. Before preparing CAS reagent, all glassware used was rinsed with 6M hydrochloric acid (HCl) to remove any traces of iron and washed five to seven times in deionized water. In a 96-well microplate, 100 μL of CAS reagent was added to 100 μL of each bacterial supernatant. After incubation in the dark for 2 h, absorbance was measured at 630 nm. Each sample was analyzed in three technical replicates. Siderophore production was expressed as percent siderophore units (PSU), calculated using the following Equation [2]:


$$\begin{array}{l} PSU=\;\left[\left(Ar-As\right)/Ar\right]\times 100 \end{array}$$ (2)

where *Ar* is the absorbance of the reference (CAS reagent alone) and *As* is the absorbance of the sample at 630 nm.

#### 2.4.7 Motility assays

To evaluate the ability of bacteria to move independently using metabolic energy, a motility assay was performed as previously described by Martínez-Cámara et al. (2020). Briefly, bacteria were grown in LB medium at 28°C for 24 h. After incubation, fresh colonies were inoculated on 5 mL of LB medium at 28°C and 180 rpm for 24 h. Then, 5 μL of bacterial suspensions (OD_600_ ≈ 0.3) were dropped in the center of each plate. Petri dishes were prepared with LB plus 0.3% (w/v) of bacteriological agar, for swimming motility. The plates were incubated for 24 h at 28 °C. The extent of motility was determined by measuring the diameters of migration halos, and the results expressed in cm of bacterial extension on the media. Only swimming motility was assessed in this study, as it is commonly associated with bacterial dispersal and early root colonization.

#### 2.4.8 pH, salt and osmotic stress tolerance

The tolerance to acidic and alkaline pH was assessed in LB medium adjusted to pH 4, 5, 7 (control), and 8, following the methodology of Lopes et al. (2024). Salt tolerance was evaluated according to Zahra et al. (2023), with minor modifications. Briefly, LB medium was supplemented with 0% (control), 2.5%, 5%, and 10% (w/v) NaCl. pH adjustment was performed using 1 M HCl or NaOH. In both assays, isolates were grown in LB medium at 28 °C and 180 rpm overnight, and the optical density (OD600) was adjusted to approximately 0.1. Subsequently, 10 μL of each isolate was inoculated into 200 μL of the respective medium (with different pH values or salt concentrations) in 96-well plates, in triplicate, and incubated at 28 °C and 130 rpm for 24 h.

The tolerance to water stress was tested in LB medium with different water potentials calculated according to the Equation [3] (0 Mpa, −0.05 Mpa, −0.15 Mpa, −0.49 Mpa, and −1.03 MPa). This was prepared by adding 0, 5, 10, 20 and 30 g of polyethylene glycol (PEG-4000), respectively, in 100 mL of LB, following the protocol adapted from Busse and Bottomley (1989). After overnight culture, the OD600 was adjusted to approximately 0.4, and 10 μL of each culture was inoculated into 5 mL of LB medium containing the respective PEG concentration. No supplemented LB medium was used as a control. Cultures were incubated at 28 °C and 180 rpm for 24 h. The OD600 was measured using a Multiskan SkyHigh Microplate Spectrophotometer (Thermo Fisher Scientific™, USA). Absorbance values were compared with the control to evaluate the tolerance of the isolates to osmotic stress.


$$\begin{array}{l} A=\Psi (bars)=-[1.18\times 10^{-2}+1.18\times 10^{-4}C^{2}\\ \;+2.67\times 10^{-4}CT+8.39\times 10^{-\;7}C^{2}T] \end{array}$$ (3)

Where T = room temperature (25°C) and C = PEG concentration (g Kg^−1^ H_2_0), 1 bar = 0.1 MPa

### 2.5 Effects of bacterial inoculation on early growth of wheat seedlings

**Figure 1:**
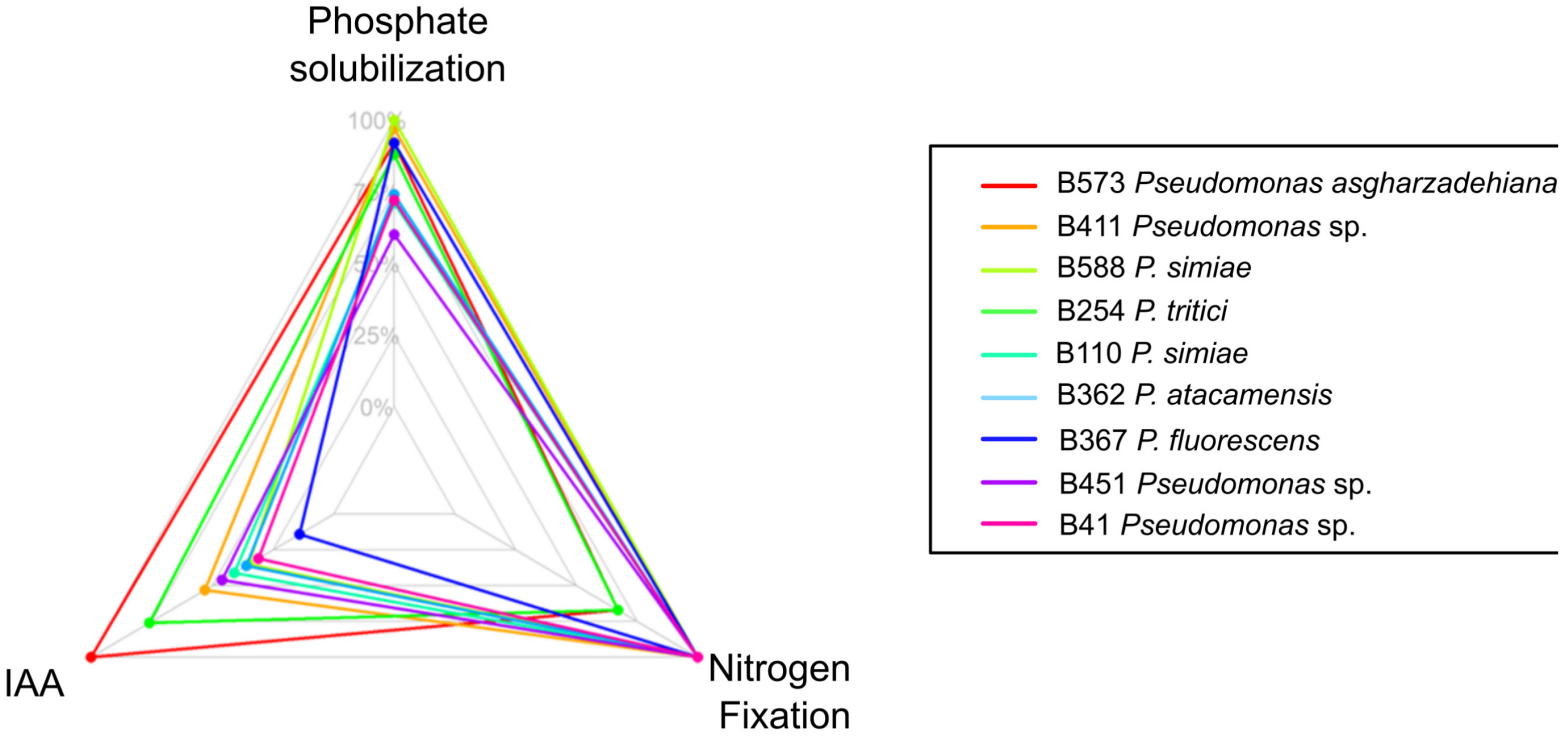
Ternary plot of the best-performing isolates of the family *Pseudomonadaceae* based on normalized data using Min-Max Scaling (linear normalization to the range) based on three plant growth-promoting traits: phosphate solubilization index (PSI), production of Salkowski-reactive indolic compounds, and presumptive nitrogen-fixing potential.

**Figure 2:**
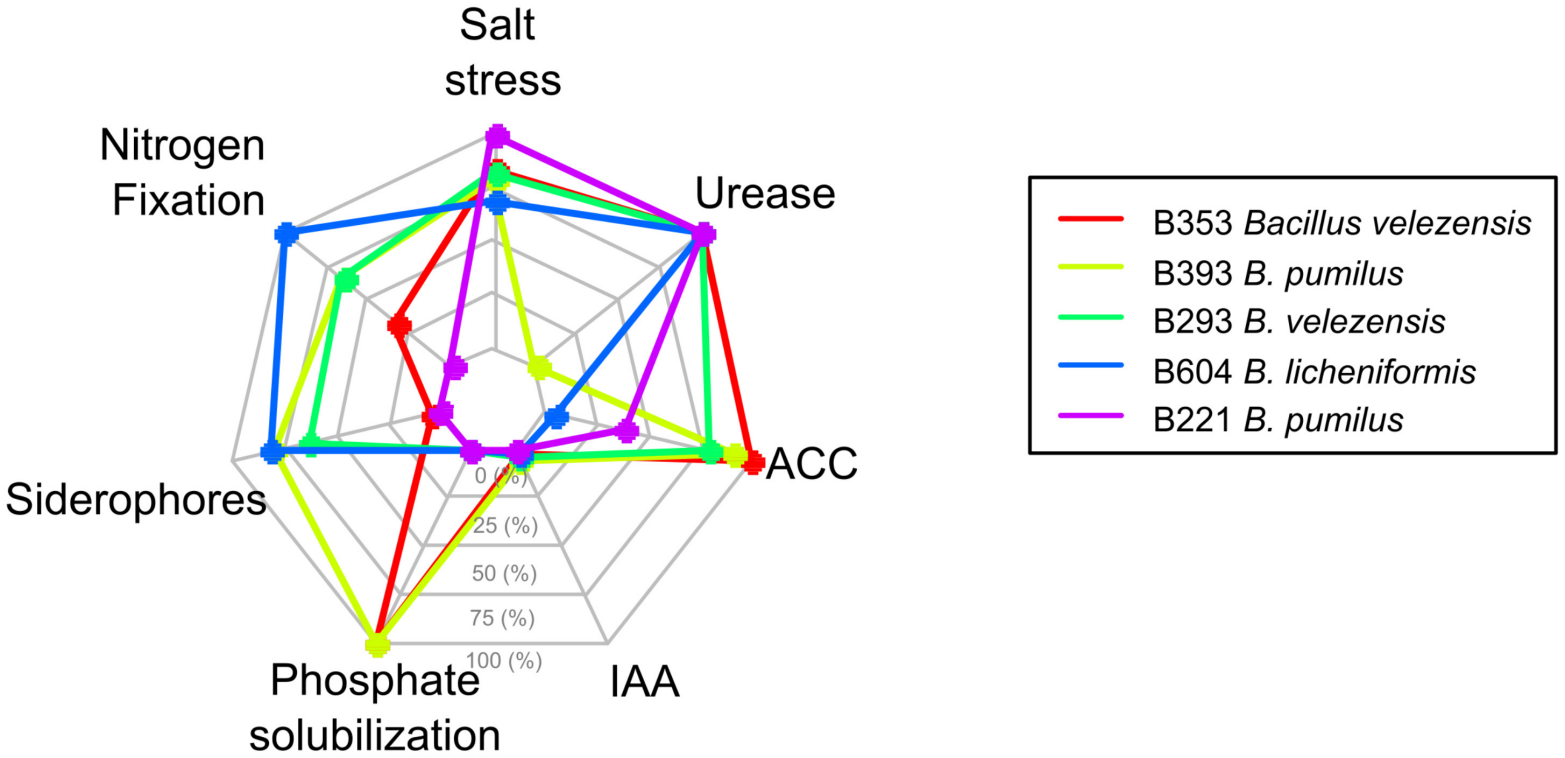
Radar plot of the best-performing bacilli isolates based on normalized data using Min-Max Scaling (linear normalization to the range) based on seven evaluated plant growth-promoting traits: phosphate solubilization index (PSI), production of Salkowski-reactive indolic compounds, presumptive nitrogen-fixing potential, salinity tolerance, siderophore production, ACC deaminase activity, and urease activity.

Isolate selection for germination and early seedling development assays was based on a composite performance score calculated from Min-Max normalized datasets (linear transformation to a 0–1 range) integrating three initial plant growth-promoting traits: phosphate solubilization, IAA-equivalent production, and presumptive nitrogen fixation. Equal weighting was applied to each trait. To improve transparency and facilitate reproducibility of the selection procedure, the normalized values used for score calculation is provided in Supplementary Table S5. An initial screening strategy was applied to 43 *Pseudomonas* isolates, for which these three traits provided sufficient variation to rank candidates. The highest-scoring isolates were selected for subsequent functional characterization and seedling bioassays (Figure 1). In contrast, *Bacillaceae* and *Paenibacillaceae* isolates showed limited discrimination based on the initial screening, with most strains exhibiting low or absent phosphate solubilization and indole compound production, whereas presumptive nitrogen fixation showed greater variability (Supplementary Table S4). Therefore, considering the smaller number of isolates and the limited discriminatory power of the initial screening, all *Bacillaceae* and *Paenibacillaceae* isolates were retained and evaluated using a broader functional profile based on seven plant growth-promoting and stress-related traits (Figure 2). Data normalization was performed using the vegan package v.2.7.1 (Oksanen et al., 2024), and radar plots were generated using the fmsb package v.0.7.6 (Nakazawa, 2024). All analyses were conducted in RStudio v.4.5.0 (Rstudio Team, 2025).

#### 2.5.1 Bacterial inoculum preparation

The selected bacteria were initially grown in LB agar plates at 28 °C for 24 h to obtain well-developed colonies. A single colony of each isolate was then transferred to 5 mL LB broth in 50 mL falcon tubes and incubated at 28 °C on a rotary shaker at 180 rpm. After 3 days, each bacterial culture was pelleted at 5,000 × g for 10 min and resuspended in distilled water. The optical density of each bacterial suspension was measured with a spectrophotometer (Multiskan SkyHigh Thermo Fisher Scientific™, USA) and adjusted to an OD_600_ = 0.5.

#### 2.5.2 Wheat seed sterilization and priming

Wheat seeds of the cultivar Tocayo were first surface sterilized in 1% (v/v) sodium hypochlorite (NaClO) solution for 2 min, followed by 2 min immersion in 70% (v/v) ethanol and then rinsed five times in sterile deionized water under a laminar flow (2 min per rinse), following a protocol stablished by Sauer and Burroughs (1986). The surfaced-sterilized seeds were then dipped into the bacterial suspended in distilled water for 45 min and air-dried aseptically in the laminar air flow for 15 min before planting. The control group comprised surface sterilized seeds, immersed in sterilize distilled water.

#### 2.5.3 Experimental design and growth conditions

Solid medium for *in vitro* germination was prepared following the descriptions of Azaroual et al. (2020), with some modifications. Briefly, phytoagar (7 g L^−1^) was dissolved in ultrapure water and the pH adjusted to 5.8 with 1 M KOH/HCl prior to sterilization. The medium was autoclaved, dispensed aseptically as 10 mL per sterile glass jar in a laminar-flow cabinet and allowed to solidify. Seven seeds, previously sterilized and soaked into the respective bacterial suspensions were placed in each jar and incubated at 25 °C for 72 h in the dark. Non-inoculated seeds (soaked in distilled water) served as control. Each treatment included four replicates. After the emergence of the radicle, the jars were transferred to a climate chamber (Fitoclima 1200, Aralab, Portugal) set at 25/20 °C (day/night), 16-h light/8-h dark photoperiod, and 70% relative humidity for 4 days. At the end of the experiment, the germination rate, number of roots, shoot and root lengths, and shoot and root fresh and dry weights were recorded. Fresh and dry biomass were measured individually for each germinated seedling after separating shoots and roots. Mean biomass per seedling was then calculated for each replicate based exclusively on germinated seedlings. Afterwards, the tissues were dried at 70 °C for 48 h in a forced-air oven until constant weight was reached, and the dry weights of shoots and roots were determined.

### 2.6 Statistical analysis

All data were processed using IBM SPSS v.30 (SPSS Inc., Chicago, USA). Descriptive statistics (mean ± standard deviation) were calculated for all quantitative variables. For the selection of the most promising isolates, data were normalized using Min-Max Scaling (linear normalization to the range 0–1). Heatmap and plots were generated in RStudio v.4.5.0 (Rstudio Team, 2025). One-way ANOVA followed by Tukey's honestly significant difference (HSD) test (*p* < 0.05) was applied only to experiments with replicated quantitative measurements, namely the biochemical assays (pH, salt and osmotic stress tolerance), and the wheat seedling bioassays. In contrast, the initial screening of plant growth-promoting traits (phosphate solubilization, siderophore production, ACC deaminase activity, nitrogen-free medium growth, and indolic compound production) was intended as a descriptive characterization of the isolate collection. Therefore, these screening data are presented using descriptive statistics only, and no inferential comparisons among isolates were performed.

## 3 Results

### 3.1 Isolation and molecular characterization

Wheat plant organs (roots, leaves, and grains) collected from seven wheat fields, across distinct agroecosystems, yielded 181 endophytic isolates belonging to the families *Pseudomonadaceae, Bacillaceae*, and *Paenibacillaceae* (Supplementary Table S1). Overall, roots yielded the highest number of isolates (91; 50.3%), followed by leaves (66; 36.5%) and grains (24; 13.3%). *Pseudomonadaceae* was the most abundant family, comprising 127 isolates (70.2%), and was recovered from all plant compartments, with the greatest proportion isolated from leaves (58 isolates; 45.7% of *Pseudomonadaceae* isolates), followed by roots (51; 40.2%) and grains (18; 14.2%). Likewise, *Bacillaceae* (18 isolates; 9.9%) and *Paenibacillaceae* (36 isolates; 19.9%) were predominantly recovered from roots, accounting for 77.8% and 72.2% of isolates within each family, respectively. *Pseudomonadaceae* were also particularly abundant in the conventional irrigated field and the light medium clay soil, which accounted for 14.4% and 26.5% of the total isolates, respectively (Supplementary Table S1).

To reduce redundancy, the 181 isolates were characterized using BOX-PCR fingerprinting, from which a representative set of 66 isolates (43 *Pseudomonadaceae*, 11 *Bacillaceae*, and 12 *Paenibacillaceae*) was selected for further molecular identification using multilocus sequence analysis. A preliminary taxonomic assignment was performed using BLASTn of 16S rRNA gene sequences, assigning the isolates to the genera *Bacillus, Paenibacillus, Peribacillus, Terribacillus, Solibacillus, Psychrobacillus, Pseudomonas*, and *Phytopseudomonas*. For the family *Pseudomonadaceae*, the concatenated dataset of 16S rRNA, *gyrB*, and *rpoD* genes comprised 162 ingroup taxa and one outgroup taxon, including 43 sequences generated in this study and 116 retrieved from GenBank, with a total alignment length of 2,680 characters (1,416 from 16S rRNA, 614 from *gyrB*, and 650 from *rpoD*, including gaps). For the families *Bacillaceae* and *Paenibacillaceae*, the combined 16S rRNA and *gyrB* dataset included 62 ingroup taxa (23 sequences from this study and 39 from GenBank) and comprised 1,940 characters (1,536 from 16S rRNA and 404 from *gyrB*, including gaps). Phylogenetic analyses placed the *Pseudomonadaceae* isolates into 31 clades corresponding to 17 *Pseudomonas* species, two *Phytopseudomonas* species, and 12 unidentified *Pseudomonas* species (Supplementary Figure S1). Isolates assigned to the families *Bacillaceae* and *Paenibacillaceae* clustered into 11 clades corresponding to 10 known species and one unidentified *Solibacillus* species (Supplementary Figure S2). Within the ML phylogeny of the family *Pseudomonadaceae*, the genus *Pseudomonas* was represented by seven major subgroups, with the *P. fluorescens* group containing the highest number of isolates (23 out of 43; 53%), followed by the *P. mandelii, P. corrugata, P. chlororaphis, P. koreensis, P. lutea*, and *P. putida* groups. Regarding the families *Bacillaceae* and *Paenibacillaceae*, the species *Paenibacillus amylolyticus* was the most abundant species (8 out of 23; 35%).

### 3.2 Biochemical characterization for PGPB traits

#### 3.2.1 Indole compounds production

Production of indole compounds, expressed as IAA-equivalents, was detected in all isolates (n = 66), with concentrations ranging from 0.06 to 143.56 μg mL^−1^. Overall, 63 isolates (95.5%) were considered positive for indole compounds production. The highest IAA-equivalents concentration was observed in isolates B254 (*P. tritici*), B505 (*P. shirazensis*), B573 (*P. asgharzadehiana*), and B588 (*P. simiae*), producing 99.2, 109.6, 114.6, and 143.6 μg mL?^1^, respectively (Supplementary Table S4).

#### 3.2.2 Phosphate solubilization

A total of 66 isolates were evaluated for phosphate solubilization on Pikovskaya medium, of which 62% showed positive activity, with halo diameters ranging from 1.08 to 3.21 mm. Among these, 11 isolates, all belonging to the genus *Pseudomonas*, exhibited PSI values greater than 2 mm. These included isolates B153, B160, B155, B41, B411 (*Pseudomonas* sp.) B110 (*P. simiae*), B362 (*P. atacamensis*), B254 (*P. tritici*), B367 (*P. fluorescens*), B573 (*P. asgharzadehiana*), and B588 (*P. simiae*), with isolate B588 showing the highest PSI value. In contrast, isolates belonging to the families *Bacillaceae* and *Paenibacillaceae*, showed lower phosphate-solubilizing activity, with only six isolates testing positive and halo diameters ranging from 1.08 to 1.30 mm (Supplementary Table S4).

#### 3.2.3 Potential nitrogen fixation

Presumptive nitrogen-fixing ability, as determined by growth and bromothymol blue color change in nitrogen-free medium, was observed in 68.2% of the isolates. Among these putative diazotrophic isolates, 86.7% belonged to the family *Pseudomonadaceae*, whereas *Paenibacillaceae* and *Bacillaceae* accounted for 8.9% and 4.4%, respectively (Supplementary Table S4).

#### 3.2.4. Selection of candidate isolates for further analyses

Based on these results, nine *Pseudomonas* strains were identified as the most promising candidates, thereby reducing both the number of isolates and experimental redundancy. This selection was supported by a heat map (Supplementary Figure S3) and a ternary plot analysis integrating the three traits to rank strain performance (Figure 1). However, these assays provided limited discriminatory power for isolates belonging to the *Bacillaceae* and *Paenibacillaceae* families, as the measured traits displayed consistently low values with little variation among isolates (Supplementary Table S4). Consequently, subsequent analyses were conducted on all 23 isolates belonging to these families, in addition to the nine selected *Pseudomonas* strains.

#### 3.2.5. ACC deaminase

ACC deaminase activity was observed in all isolates, ranging from 33.6 to 48.8 μmol mL^−1^. Notably, isolates B293, B353 (*B. velezensis*) and B393 (*B. pumilus*) exhibited the highest ACC deaminase activities, with values of 45.7, 47.3 and 48.8 μmol mL^−1^, respectively (Supplementary Table S4).

#### 3.2.6. Siderophore production

Siderophore production was detected in all isolates, with percent siderophore unit (PSU) values ranging from 0.93% to 48.99%. Isolates B573 (*P. asgharzadehiana*) and B487 (*B. licheniformis*) exhibited the highest siderophore production, reaching 45.15 and 48.99%, respectively. In contrast, isolates B221 (*B. pumilus*), B194 and B338 (*Paenibacillus amylolyticus*), and B437 (*Solibacillus* sp.) showed minimal production, with values below 2% (Supplementary Table S4).

#### 3.2.7. Urease activity

Regarding urease activity, all the *Pseudomonas* isolates were positive (*n* = 9), apart from isolate B110 (*P. simiae*). On the other hand, only 26% of *Bacillaceae* and *Paenibacillaceae* isolates demonstrated urease activity, including isolates B192 (*Terribacillus saccharophilus*), B198 (*Paenibacillus frigorotolerans*), B201 (*P. nuruki*), B221 (*B. pumilus*), B353 (*B. velezensis*) and B598 (*P. amylolyticus*) (Supplementary Table S4).

#### 3.2.8. Bacterial motility

Motility varied among isolates, with diameters ranging from 14.48 to 90.00 mm in semi-solid medium (0.3% agar) (Supplementary Table S4). On average, isolates belonging to the *Bacillaceae* and *Paenibacillaceae* families (*n* = 23) showed higher motility compared to the genus *Pseudomonas*.

#### 3.2.9. pH, salt and osmotic stress tolerance

Tolerance assays were conducted to evaluate bacterial growth under osmotic, salinity and pH stress. Regarding pH, all isolates (n = 32) exhibited robust growth at pH valued from 5 to 8, whereas growth was largely inhibited at pH 4. The exceptions were isolates B221 (*B. pumilus*), B293 (*B. velezensis*) and B598 (*Paenibacillus amylolyticus*), all of which showed tolerance to acidification (pH 4). Medium at pH 5 significantly promoted growth of isolate B588 (*Pseudomonas simiae*) (*p* < 0.001), with a 7% increase relative to the control. Moreover, pH 8 enhanced the growth of isolates B437 (*Solibacillus* sp.) (*p* < 0.001) and B602 (*Paenibacillus amylolyticus*) (*p* = 0.003), with increases of 11.4% and 35%, respectively (Supplementary Table S6).

NaCl at 2.5% (w/v) stimulated bacterial growth compared to the control in all tested isolates. Overall, of the 32 tested isolates, 40.6% tolerated NaCl concentrations of up to 5% (w/v) (Supplementary Table S7). Increasing salinity to 10% NaCl inhibited the growth of most isolates. However, 12.5% (isolates B192 *T. saccharophilus*, B338 *Paenibacillus amylolyticus*, B353 *B. velezensis* and B437 *Solibacillus* sp.), all members of the families *Bacillaceae* and *Paenibacillaceae*, were able to tolerate this high salt concentration.

In the water stress assays, most isolates exhibited a significant reduction in growth in the presence of PEG 4000, with a marked decline at 20–30% concentrations. Isolates B76 (*p* < 0.001), B527 (*p* < 0.001), B598 (*Paenibacillus amylolyticus*) (*p* < 0.001) and B411 (*Pseudomonas* sp.) (*p* < 0.001), showed tolerance to PEG-induced osmotic stress up to 5% PEG 4000. Notably, isolate B598 (*P. amylolyticus*) (*p* < 0.001) demonstrated enhanced tolerance, maintaining growth at concentrations up to 10% PEG 4000 (Supplementary Table S8).

### 3.3 Effects of bacterial inoculation on wheat seedlings performance

Following the expanded set of biochemical assays, we refined the selection to identify the most promising strains within the *Bacillaceae* and *Paenibacillaceae* families using a radar plot integrating multiple key traits, including IAA production, phosphate solubilization, siderophore production, ACC deaminase activity, nitrogen fixation, and salinity tolerance. Based on this multivariate analysis, the five top-performing isolates were selected (Figure 2). Subsequently, these five isolates, together with the nine previously selected *Pseudomonas* strains, were then tested for their effect on germination and early growth of wheat seedlings.

**Figure 3:**
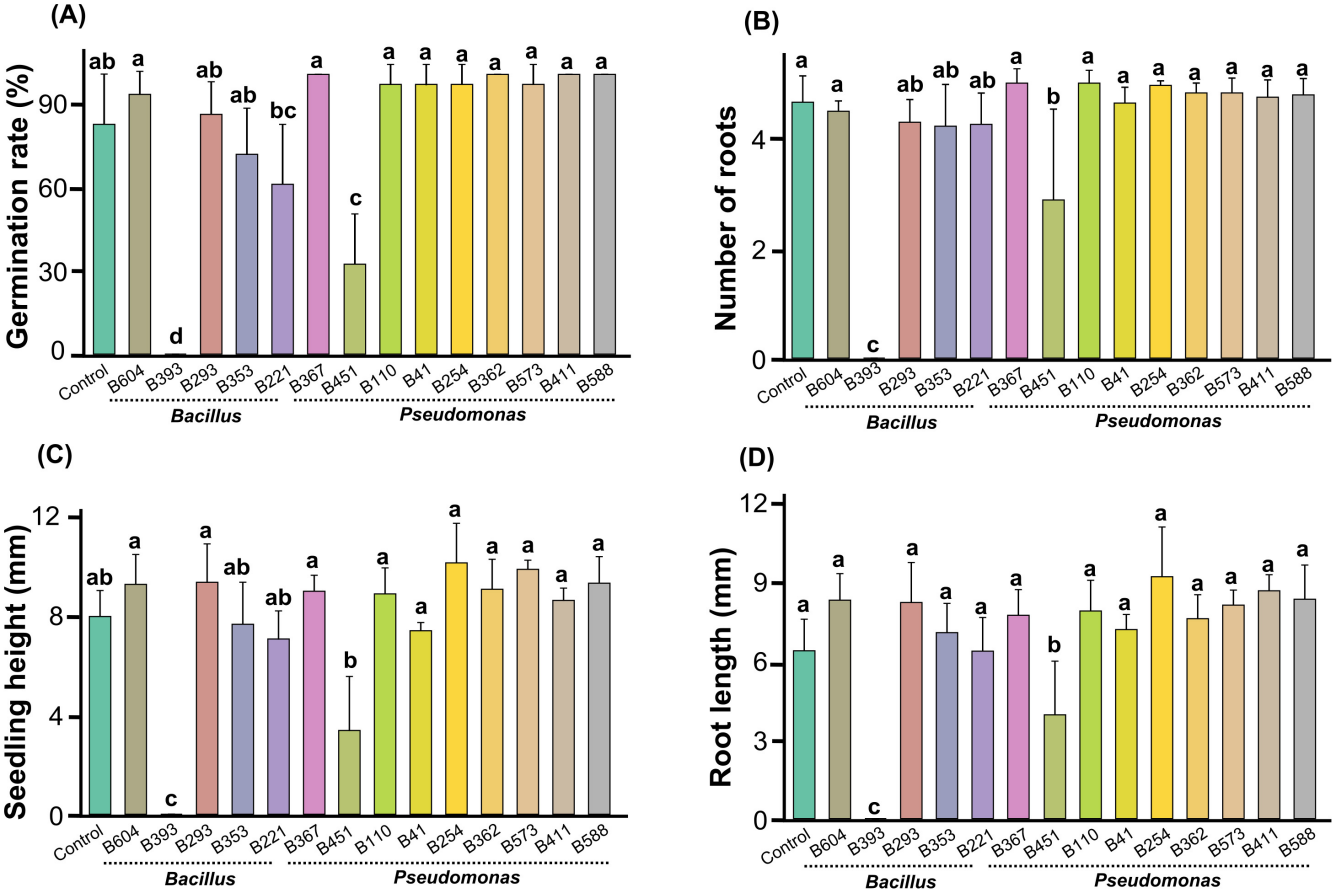
Effects of isolates from the genera *Bacillus* and *Pseudomonas* on wheat seedling performance: germination rate (%) **(A)**, number of roots **(B)**, seedling height (mm) **(C)** and root length (mm) **(D)** at early growth stage. Values represent means, and bars represent standard deviation. Different letters indicate significant differences among treatments according to Tukey's test at 5% significance level. Identification of the isolates is given in Supplementary Table S4.

**Figure 4:**
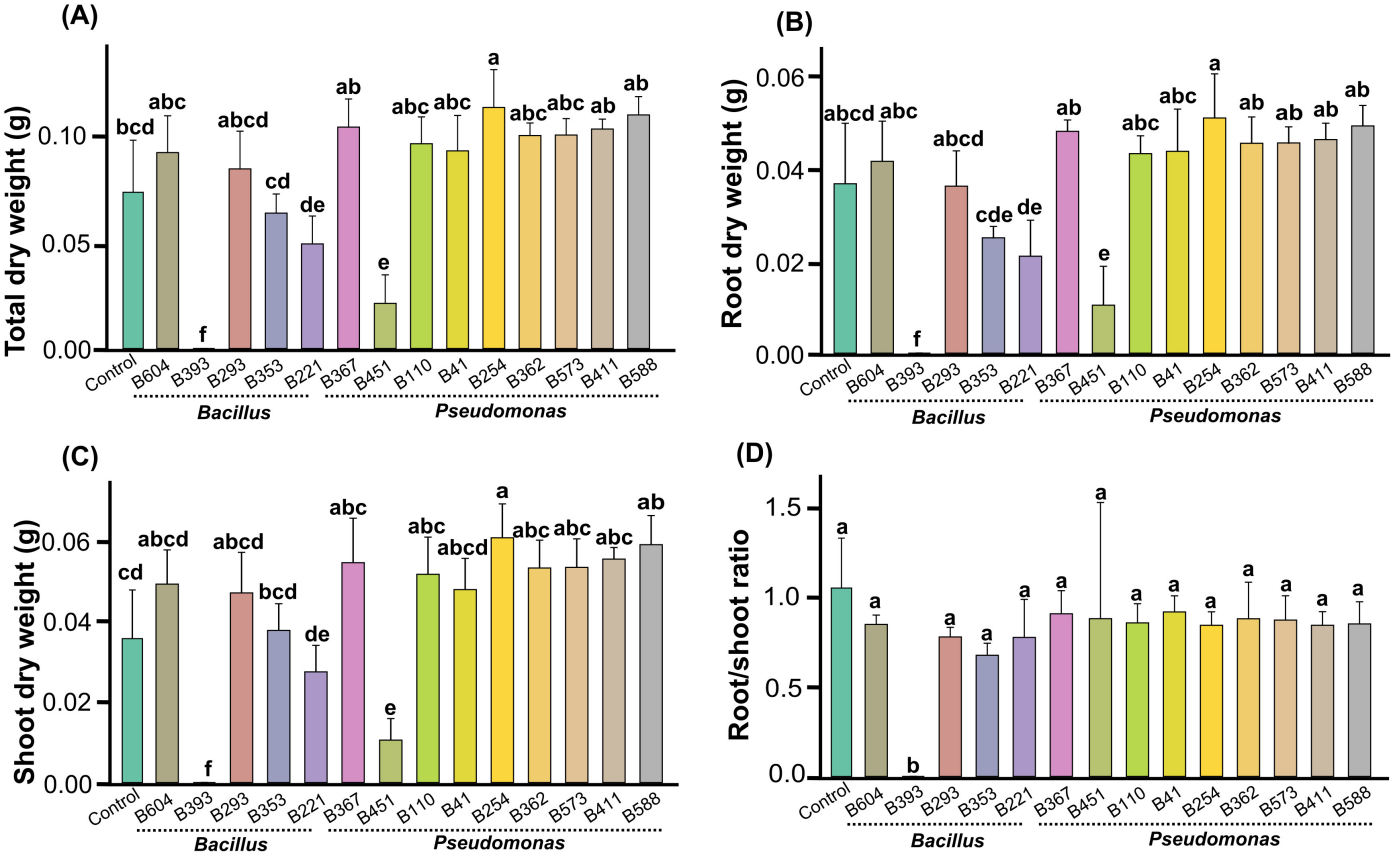
Effects of isolates from the genera *Bacillus* and *Pseudomonas* on wheat seedling performance: total dry weight (g) **(A)**, root dry weight (g) **(B)**, shoot dry weight (g) **(C)** and root/shoot ratio **(D)** at early growth stage. Values represent means, and bars represent standard deviation. Different letters indicate significant differences among treatments according to Tukey's test at 5% significance level. Identification of the isolates is given in Supplementary Table S4.

Bacterial inoculation significantly affected germination, early seedling development, and biomass accumulation (Figures 3, 4), with marked variability among isolates. Germination rates (Figure 3A) were negatively affected by inoculation with certain isolates. In particular, B393 (*Bacillus pumilus*) completely inhibited germination, whereas B451 (*Pseudomonas sp*.) significantly reduced germination to 60.9%. The number of roots per seedling (Figure 3B) was generally comparable to the control across treatments. However, inoculation with B393 (*Bacillus pumilus*) completely abolished root formation, while B451 (*Pseudomonas sp*.) significantly reduced root number, reaching values 31.8–42.5% lower than those observed for most other isolates.

Seedling height and root length exhibited comparable responses to bacterial inoculation (Figures 3C, D). Inoculation with B451 (*Pseudomonas sp*.) significantly reduced seedling height and root length by 38.2% and 57.6%, respectively, compared with the control. In contrast, seeds treated with B393 (*Bacillus pumilus*) failed to germinate, resulting in a complete inhibition (100%) of both shoot and root development.

Biomass accumulation reflected the patterns observed for early growth (Figure 4). Since biomass was calculated on a per-germinated-seedling basis, the reported values are independent of differences in germination percentage among treatments. Total dry weight (Figure 4A) was significantly higher in seedlings inoculated with B254 (*P. tritici*), showing an increase of 35.2%. This response was driven by gains in both root dry weight (Figure 4B) and shoot dry weight (Figure 4C), with B254 producing the highest root biomass values among all treatments. The root/shoot ratio (Figure 4D) remained largely stable across most inoculated treatments, suggesting that bacterial inoculation promoted overall biomass accumulation without altering the balance between above- and belowground growth. Exceptions were observed for B393 (*B. pumilus*), which exhibited a markedly reduced ratio reflecting the near-complete absence of seedling development.

## 4 Discussion

This study presents a diverse and functionally rich collection of wheat endophytes isolated from different agroecosystems. By combining cross-regional sampling with taxonomic and functional screening, we increased the likelihood of identifying strains with broad ecological tolerance and agronomic relevance, an approach increasingly recommended for bioinoculant discovery (Timmusk et al., 2023; Salem et al., 2024; Valente et al., 2025). The dominance of pseudomonads among isolates from irrigated roots is consistent with previous studies showing that increased soil moisture favors copiotrophic taxa, including *Pseudomonas*, capable of rapidly exploiting water availability (Li et al., 2021; Garrido-Sanz et al., 2023). Overall, roots yielded the highest number of endophytic isolates, followed by leaves and grains. *Pseudomonadaceae* were recovered from all plant compartments, whereas *Bacillaceae* and *Paenibacillaceae* were mainly associated with roots. However, whether tissue of origin predicts functional traits or plant growth-promoting performance remains to be determined and could be an important avenue for future research. Although bacilli were less frequently isolated in the current study, their presence remains agronomically important, as spore-forming bacteria are often underrepresented in sequencing surveys but are particularly attractive as inoculants due to their persistence under unfavorable conditions (Dasila et al., 2023; Patel et al., 2023).

Beyond taxonomic diversity, the functional profiling of bacterial isolates provides key insights into their potential as bioinoculants (Jakubowska et al., 2025). Traits associated with plant growth promotion, together with the ability to tolerate abiotic stresses such as extreme pH, salinity, and osmotic stress, are critical determinants of inoculant establishment, persistence, and efficacy under field conditions (Igiehon et al., 2025; Kaya et al., 2026). Since agricultural environments are inherently heterogeneous and frequently exposed to environmental constraints, the identification of strains combining multiple beneficial functions with broad stress tolerance is expected to increase the likelihood of successful application across diverse agroecosystems (Igiehon et al., 2025; Jakubowska et al., 2025). Therefore, evaluating both plant growth-promoting traits and stress resilience represents an essential step toward the development of robust and reliable microbial inoculants (Kaya et al., 2026).

### 4.1 Multifunctional traits support the plant growth-promoting potential of wheat endophytic

Among the plant growth-promoting traits assessed, phosphate solubilization stood out for its prevalence, recorded in 62% of isolates. Given wheat's high dependence on phosphate fertilization, this function is particularly significant. According to the PSI categorization (Marciano Marra et al., 2012), values above two indicate intermediate to high efficiency, placing a substantial proportion of our isolates within a functionally meaningful range. Pseudomonads consistently achieved the highest PSI values, reflecting their well-established ability to release inorganic phosphate via organic acid secretion (Amri et al., 2023), a capacity previously linked to improved soil P availability and plant uptake in wheat (Elhaissoufi et al., 2020; Dasila et al., 2023). Bacilli, by contrast, showed limited or no solubilization activity, in line with reports of comparable strains from diverse environmental origins (Santos et al., 2025). However, this apparent limitation, may be offset by other mechanisms, notably phytohormone synthesis, stress tolerance induction, and rhizosphere colonization competence (Akinrinlola et al., 2018).

Production of indole compounds, expressed as IAA-equivalents, was detected in over 95% of isolates, suggesting that auxin-related metabolic capacity is widespread within this endophytic collection. Although the Salkowski assay is widely used for preliminary screening of bacterial auxin production, it is not specific for IAA, and the detected compound may include other indole derivates. Therefore, chromatographic methods such as HPLC or LC-MS/MS could be required to confirm IAA production. Nevertheless, bacteria-auxin production has been associated with stimulation of lateral and adventitious root formation, thereby increasing root surface area and improving water and nutrients acquisition (Arzanesh et al., 2011). At the molecular level, auxin-mediated root development is regulated by complex genetic networks involving WUSCHEL-related homeobox (WOX) transcription factors, which play key roles in root meristem establishment and adventitious root formation (Abubakar et al., 2023). Although these regulatory pathways were not investigated in the present study, bacterial indolic compounds may contribute to root developmental responses through modulation of auxin-responsive genetic pathways. Pseudomonads again led in production levels, consistent with the efficiency of their tryptophan-dependent biosynthetic routes, while bacilli exhibited lower and more variable output, attributable to strain-specific differences and heightened sensitivity to culture parameters such as carbon source and incubation duration (Patten and Glick, 2002; Duca et al., 2014). Importantly, even isolates with moderate indole compounds output retain growth-promoting relevance when co-expressing traits like phosphate solubilization or ACC deaminase activity, as synergistic interactions among these functions amplify their individual effects on root architecture and nutrient uptake (Arzanesh et al., 2011; Orozco-Mosqueda et al., 2020). This synergy is further supported by the high occurrence of ACC deaminase activity across the collection. By cleaving the ethylene precursor ACC, these isolates can suppress stress-induced ethylene accumulation, thereby sustaining root elongation and overall plant performance under drought or salinity (Gupta and Pandey, 2019b). Such effects have been documented across genera: ACC deaminase-expressing *Pseudomonas* strains enhance root colonization and P solubilization under stress (Orozco-Mosqueda et al., 2020), while *Bacillus* and *Paenibacillus* counterparts improve root and shoot biomass alongside chlorophyll content (Zhao et al., 2015). The frequent co-occurrence of indole compounds production and ACC deaminase activity within individual isolates therefore points to a coordinated hormonal strategy that may collectively strengthen root development, nutrient acquisition, and, ultimately, NUE.

The detection of presumptive nitrogen-fixing ability in 68.2% of the isolates further highlights their potential to contribute to plant nitrogen nutrition. Endophytic diazotrophs have been shown to enhance nitrogen acquisition and biomass production in cereal crops (Orozco-Mosqueda et al., 2020; Guo et al., 2023) (Orozco-Mosqueda et al., 2020; Guo et al., 2023). Nevertheless, further validation using molecular or functional approaches, such as *nifH* gene detection and the acetylene reduction assay (ARA), would be required to confirm the nitrogen-fixing capacity of these isolates. Urease activity, widely observed among pseudomonads and in a proportion of bacilli, contributes to nitrogen cycling by converting urea into plant-available ammonium (Mobley and Hausinger, 1989). Considering the central role of nitrogen in wheat growth, particularly tillering (Kasemsap and Bloom, 2023), the integration of nitrogen-fixing and urease-producing bacteria may represent a sustainable strategy to improve NUE and reduce fertilizer inputs (Wang et al., 2025).

Siderophore production adds another complementary dimension to nutrient acquisition and stress tolerance. Strong siderophore activity in selected pseudomonads and bacilli aligns with their established role in iron chelation under Fe-limited conditions (Jeong et al., 2024). In wheat, siderophore-producing bacteria are associated with improved chlorophyll content, iron uptake, and enhanced tolerance to abiotic stress (Yue et al., 2022). For example, *B. subtilis* enhances wheat performance under drought through Fe-chelating compounds (Lastochkina et al., 2017), while *P. chlororaphis* O6 improves germination and stress-responsive gene expression (Yang et al., 2018).

Finally, traits related to rhizosphere competence and colonization, particularly motility, likely facilitate the effective expression of these functional mechanisms. Motility enhances chemotaxis toward root exudates and early colonization of plant tissues (Liu et al., 2024b). As demonstrated for *B. velezensis* SQR9, flagellar structures can act as adhesins, strengthening root attachment (Huang et al., 2022). Effective colonization is essential for the coordinated action of nutrient mobilization, hormone modulation, and stress alleviation (Pathak et al., 2024; Chattaraj et al., 2025). Collectively, these results suggest that the plant growth-promoting potential of wheat endophytes relies not on single traits but on the synergistic integration of nutrient acquisition, hormonal modulation, stress mitigation, and colonization capacity. Nevertheless, the effectiveness of nutrient-mobilizing bacteria also depends on host plant metabolism. Carbon allocation to root exudates, including sugars, organic acids, and phenolic compounds, shapes rhizosphere microbial activity and may influence the expression of plant growth-promoting traits, indicating that bioinoculant performance results from interactions between microbial functions and plant physiological processes (Vives-Peris et al., 2020; Chen and Liu, 2024).

### 4.2 Stress resilience as a key determinant of bioinoculant performance across agroecossystems

Abiotic stress tolerance is a fundamental criterion for the effective application of PGPB, as candidate strains must survive and remain metabolically active under adverse field conditions (Fanai et al., 2024). In wheat-based systems, drought, salinity, and pH extremes represent major constraints to productivity, particularly in Mediterranean and semi-arid regions (Lorite et al., 2023; Tita et al., 2025). Therefore, the functional relevance of traits such as phosphate solubilization, indole compounds production, nitrogen fixation, or siderophore production ultimately depends on the strain's ability to persist under stress. In this study, tolerance to pH extremes further underscores the ecological fitness of selected strains. Growth at acidic (pH 4–5) and alkaline (pH 8) conditions, particularly by *B. velezensis* B293 and *Paenibacillus amylolyticus* B598, supports previous findings that pH adaptability in PGPB is strain-specific and involves membrane and metabolic adjustments (Etesami and Maheshwari, 2018). Given that soil pH strongly regulates phosphorus availability, nitrogen transformations, and iron solubility, pH-tolerant strains are more likely to maintain functional activities such as phosphate solubilization, urease activity, and siderophore production across heterogeneous soils. Importantly, these abiotic stress tolerance traits complement colonization-related characteristics such as motility, which facilitates root attachment and early establishment. Under fluctuating environmental conditions, effective colonization ensures that multi-trait isolates can consistently deliver hormonal modulation, ethylene regulation, and nutrient mobilization.

High salt tolerance observed in several isolates aligns with previous reports demonstrating the ability of *Bacillus* spp. to grow at elevated NaCl concentrations and enhance wheat salt tolerance. For instance, Ayaz et al. (2022) demonstrated that *Bacillus* strains were able to grow up to 18% NaCl, leading to the enhancement of wheat tolerance to salt stress. Moreover, Pourbabaee et al. (2016) reported the ability of *B. mojavensis* K78 to increase wheat dry weight, as well as chlorophyll content and nutrient intake under salt stress.

Additionally, several isolates tolerated high PEG-induced osmotic stress (up to 30%), exceeding the sensitivity thresholds reported in some previous studies (Agunbiade et al., 2024), and highlighting their potential resilience under water-limiting conditions (Uzma et al., 2022). Notably, *Bacillus* and *Paenibacillus* isolates exhibited greater tolerance, consistent with their adaptive strategies, including endospore formation and structurally resilient cell envelopes (Valencia-Marin et al., 2024). Such robustness may ensure sustained expression of complementary traits under drought stress, thereby supporting root growth and nutrient acquisition when plants are most vulnerable.

### 4.3 Plant responses reveal strain-specific effects on early wheat establishment

Early developmental responses to bacterial inoculation were assessed through germination assays. Most strains promoted germination by 4–18%, though without statistical significance. However, *P. atacamensis* B362, *P. fluorescens* B367, *Pseudomonas* sp. B411, and *P. simiae* B588 achieved 100% germination rates, indicating that inoculation with these strains did not impair seed germination and was compatible with early wheat development. This observation is consistent with the well-documented role of pseudomonads in supporting plant establishment through mechanisms such as phytohormone modulation and stress alleviation. For instance, *P. protegens* DA1.2 has been shown to modulate the IAA/ABA balance and alleviate the effects of auxin-like herbicides on wheat root development (Bakaeva et al., 2022). Given the widespread occurrence of indole compounds production among the isolates evaluated in this study, hormonal regulation may have contributed to maintaining germination performance and seedling vigor.

Similarly, species of *Bacillus* have frequently been associated with improved seed performance and early seedling establishment. Biopriming studies have reported enhanced germination and radicle elongation, thereby improving water and nutrient uptake (Pérez-García et al., 2023). Moreover, *Bacillus sp*. D13 tolerated up to 30% PEG and improved wheat germination under osmotic stress (Bouremani et al., 2024), while other strains have been shown to stimulate gibberellic acid biosynthesis, promoting seedling emergence (Hu et al., 2024). These findings are consistent with the stress tolerance and multifunctional traits observed in several *Bacillus* isolates in the present study. Although the germination assay provided a useful preliminary assessment of the effects of bacterial inoculation on early wheat development, additional studies are needed to better understand the mechanisms underlying the observed responses. In particular, assessing bacterial colonization and standardizing inoculum concentrations based on viable cell counts, in addition to optical density, would strengthen the interpretation of plant-bacteria interactions. Furthermore, greenhouse and field experiments under relevant stress conditions will be necessary to determine whether these early responses translate into improved agronomic performance. Conversely, *B. pumilus* B393 and *Pseudomonas* sp. B451 inhibited and reduced germination and seedling development, respectively. Such inhibitory effects may be linked to excessive auxin production or the synthesis of phytotoxic metabolites (Etesami and Glick, 2024; Etesami, 2025). Similar negative outcomes have been reported for certain *Bacillus* and *Pseudomonas* strains in other plant systems. For example, *B. mojavensis* RRC101 and *B. amyloliquefaciens* GB03 induced phytotoxicity in *Arabidopsis thaliana* (Rath et al., 2018; Song et al., 2021), *B. megaterium* ORE8 inhibited growth in sweet pepper (Samayoa et al., 2020), and *P. brassicacearum* reduced root growth and seedling development in *Jacobaea vulgaris* (Liu et al., 2024a). While undesirable for biofertilizer development, these traits may hold potential for bioherbicidal applications (Abbas et al., 2024; Zhang et al., 2025). Nonetheless, further investigation is required to clarify the mechanisms underlying the suppressive behavior of strains B393 and B451. As the bioassays were performed using only the wheat cultivar Tocayo, cultivar-specific responses to endophytic bacteria cannot be excluded and should be investigated in future studies.

Beyond germination, root architecture is a key factor in plant tolerance to water and nutrient stress (Dobrzyński et al., 2025). However, root number and length were not significantly affected in most treatments, likely because early seedlings rely primarily on seed reserves (Irfan et al., 2024). However, *P. tritici* B254 produced significant increases in both total and shoot dry weight, suggesting a positive effect on early seedling growth under the conditions tested. This response may be associated with the strain's plant growth-promoting trait profile, including nutrient mobilization with hormonal regulation to support biomass accumulation under stress. However, the mechanisms underlying this effect were not investigated in the present study. Comparable outcomes have been documented for other *Pseudomonas* strains, including salinity-induced shoot dry weight improvements in tomato (Pandey and Gupta, 2020) and growth and biomass promotion in wheat by *P. chlororaphis* strain YB10 (*P. chlororaphis* YB10; Xu et al., 2021). Interestingly, strain B254 originated from a conventional non-irrigated field. Although its superior performance in promoting early biomass accumulation may be associated with its environmental origin, this relationship was not investigated in the present study. While this study identified several native wheat endophytes as candidates for biofertilizer development and highlighted the importance of ecological compatibility, their agronomic performance remains to be validated under greenhouse and field conditions. The introduction of non-native strains may alter resident microbial communities, as evidenced by *Streptomyces albidoflavus*, which reduced wheat endophytic actinobacterial diversity by 48% (Conn and Franco, 2004). Therefore, native strains represent promising candidates for further evaluation as biofertilizers under greenhouse and field conditions. However, their ecological compatibility and functional effectiveness should be validated across different environmental conditions before conclusions regarding local adaptation can be drawn.

## 5 Conclusions

This study expands current knowledge of wheat endophytes by characterizing a diverse collection of isolates from different agroecosystems in Spain and the Netherlands. By integrating cross-regional sampling with functional screening and seedling bioassays, we identified a broad range of functional diversity with several strains showing high plant growth-promoting potential. Pseudomonads achieved 100% seed germination and early biomass accumulation, highlighting their capacity to maintained initial plant development, in contrast, members of the *Bacillaceae* and *Paenibacillaceae* families exhibited complementary traits associated with stress mitigation and nutrient acquisition. The resilience of bacilli isolates under osmotic, saline, and pH stress, together with the functional versatility of pseudomonads, indicates that ecological adaptability and multi-trait functionality are key attributes of promising bioinoculant candidates.

Our findings further indicate that, even with robust multi-trait characterization, functional profiling alone is insufficient to reliably predict wheat performance, highlighting the complexity of plant-microbe interactions. Notably, some isolates displayed relevant plant-growth-promoting traits but inhibited germination and early seedling growth. These findings contribute to the need to combine functional profiling with plant-based evaluations when selecting microbial candidates for agricultural applications. Overall, this work emphasizes the potential of native, context-adapted wheat endophytes as candidates for sustainable biofertilization strategies. Future research should focus on validating the performance of the most promising isolates under greenhouse or field conditions, particularly under abiotic stress scenarios, to assess their capacity to improve wheat resilience, yield, and adaptation to the challenges imposed by climate change.

## Data Availability

The datasets presented in this study can be found in online repositories. The names of the repository/repositories and accession number(s) can be found in the article/supplementary material.
