# Supplementary material for: Wheat native endophytic bacteria isolated from Mediterranean and Atlantic agroecosystems: identification, functional profiling and seedling bioassays
Source: Front Microbiol. 2026 Jul 30;17:1913855. doi: 10.3389/fmicb.2026.1913855 (PMC13469647; doi:10.3389/fmicb.2026.1913855)
Supplement: Supplementary file 1 [file Table_1.docx]

**Table S1**. Sampling regions in Spain (SP) and the Netherlands (NL). and the number of isolates obtained. in each type of plant organ. belonging to the families *Pseudomonadaceae* (*Pseud.)*. *Bacillaceae (Bacill.)*. and *Paenibacillaceae (Paenibacill.)*.

.

| **Country** | **Region** | | **Agricultural practice / soil type** | **Source** | **Isolates obtained** | | |  |
| --- | --- | --- | --- | --- | --- | --- | --- | --- |
|  |  |  |  |  |  |  |  |  |
|  |  |  |  |  | *Pseud.* | *Bacill.* | *Paenibacill.* |  |
| SP | Zamora | | Conventional Irrigated | Roots | 11 (6.1%) | 4 (2.2%) | 3 (1.7%) |  |
|  |  |  |  | Leaves | 10 (5.5%) | 1 (0.6%) | 2 (1.1%) |  |
|  |  |  |  | Grain | 5 (2.8%) | 0 | 3 (1.7%) |  |
|  |  |  | Conventional non-irrigated | Roots | 5 (2.8%) | 0 | 10 (5.5%) |  |
|  |  |  |  | Leaves | 6 (3.3%) | 2 (1.1%) | 0 |  |
|  |  |  |  | Grain | 7 (3.9%) | 0 | 0 |  |
|  |  |  | Organic non-irrigated | Roots | 4 (2.2%) | 6 (3.3%) | 9 (5.0%) |  |
|  |  |  |  | Leaves | 10 (5.5%) | 0 | 2 (1.1%) |  |
|  |  |  |  | Grain | 0 | 0 | 0 |  |
| NL | Lelystad | | Light medium clay soil | Roots | 31 (17.1%) | 4 (2.2%) | 4 (2.2%) |  |
|  |  |  |  | Leaves | 12 (6.6%) | 0 | 0 |  |
|  |  |  |  | Grain | 5 (2.8%) | 0 | 0 |  |
|  | Westmaas | | Heavy clay | Roots | 0 | 0 | 0 |  |
|  |  |  |  | Leaves | 9 (5.0%) | 0 | 0 |  |
|  |  |  |  | Grain | 0 | 0 | 0 |  |
|  | Valthermond | | Peatsand | Roots | 0 | 0 | 0 |  |
|  |  |  |  | Leaves | 6 (3.3%) | 1 (0.6%) | 2 (1.1%) |  |
|  |  |  |  | Grain | 0 | 0 | 0 |  |
|  | Nieuw Beerta | | Very heavy clay | Roots | 0 | 0 | 0 |  |
|  |  |  |  | Leaves | 5 (2.8%) | 0 | 1 (0.6%) |  |
|  |  |  |  | Grain | 1 (0.6%) | 0 | 0 |  |
|  | | Total | |  | 127 (70.2%) | 18 (9.9%) | 36 (19.9%) |  |

**Table S2**. List of pseudomonads and bacilli used in this study for phylogenetic analysis.

| Species | Strain | Country | Host/Environment | Genome Accession | 16S rRNA | *rpoD* | *gyrB* |
| --- | --- | --- | --- | --- | --- | --- | --- |
| *Azotobacter salinestris* | **KACC 13899** | South Korea | soil | CP045302 | - | - | - |
| *Bacillus licheniformis* | DSM 13 | Germany | biocompost | CP093290 | - | - | - |
|  | KNU11 | South Korea | soil | CP042252 | - | - | - |
|  | 14ADL4 | South Korea | High-salt fermented soybean | CP026673 | - | - | - |
|  | **ATCC 14580** | India | tannery effluent sample | CP140161 | - | - | - |
| *Bacillus pumilus* | UAMX | Mexico | *Homo sapiens* | CP058951 | - | - | - |
|  | **NBRC 12092** | USA | soil | JALJWW000000000 | - | - | - |
|  | X14-67 | China | natural aged pericarps | CP170098 | - | - | - |
| *Bacillus velezensis* | B1 | China | *Dendrobium huoshanense* | CP097895 | - | - | - |
|  | CBMB205 | South Korea | *Oryza sativa* | CP011937 | - | - | - |
|  | **NRRL B-41580** | Spain | river Velez | LLZC00000000 | - | - | - |
| *Paenibacillus amylolyticus* | SQR-21 | Canada | *Triticum turgidum* | CP107037 | - | - | - |
|  | **NRS-290** | USA | soil | BIMJ00000000 | - | - | - |
|  | YS5-7 | Canada | soil | CP145892 | - | - | - |
|  | Cl19 | USA | gastrointestinal tract | CP168019 | - | - | - |
|  | PAE4 | China | soil | CP158833 | - | - | - |
| *Paenibacillus lautus* | **NBRC 15380** | - | Intestinal tract of child | BIMF00000000 | - | - | - |
|  | E7593-69 | USA | Tattoo ink | CP032412 | - | - | - |
| *Paenibacillus nuruki* | **NBRC 112013** | South Korea | Korean traditional nuruk | MDER00000000 |  |  |  |
|  | AASFL403 | Bulgaria | *Astragalus aitosensis* | OY725677 | - | - | - |
| *Paenibacillus pabuli* | **NRS-924** | - | green or fermenting fodder | JARLKW000000000 |  |  |  |
|  | E1 | China | soil | CP073714 | - | - | - |
| *Paenibacillus terrae* | HPL-003 | South Korea | soil | CP003107 | - | - | - |
| *Paenibacillus tundrae* | DS1314 | - | soil | JAUSTI000000000 | - | - | - |
| *Paenibacillus xylanilyticus* | **LMG 21957** | Spain | laboratory air | JABMCB000000000 | - | - | - |
| *Peribacillus frigoritolerans* | LN4 | China | soil | CP128118 | - | - | - |
|  | **DSM 8801** | Morocco | soil | JALJWT000000000 | - | - | - |
|  | Q2H1 | China | potato root | CP110132 | - | - | - |
|  | CK6 | China | soil | CP085395 | - | - | - |
| *Phytopseudomonas argentinensis* | **LMG 22563** | Argentina | *Chloris ciliata* | FORC00000000 | - | - | - |
| *Phytopseudomonas flavescens* | **LMG 18387** | USA | *Juglans regia* | FNDG00000000 | - | - | - |
| *Phytopseudomonas punonensis* | **DSM 27507** | Peru | *Poaceae* plants | FRBQ00000000 | - | - | - |
| *Phytopseudomonas seleniipraecipitans* | **LMG 25475** | USA | soil | FNBM00000000 | - | - | - |
| *Phytopseudomonas straminea* | **LMG 21615** | Japan | *Oryza sativa* | FOMO00000000 | - | - | - |
|  | NBRC 16640 | Japan | *Oryza sativa* | BSSG00000000 | - | - | - |
| *Pseudomonas allii* | **MAFF 301514** | Japan | *Allium cepa* | JABUHT000000000 | - | - | - |
| *Pseudomonas alvandae* | **DSM 112525** | Germany | soil sample bean agriculture | JAEKIK000000000 | - | - | - |
| *Pseudomonas alvandae* | **SWRI17** | Iran | *Triticum aestivum* | CP077080 | - | - | - |
| *Pseudomonas antarctica* | **CMS 35** | Antarctica | Cyanobacterial mat samples | JXDI01000000 | - | - | - |
| *Pseudomonas asgharzadehiana* | Phyllo_192 | USA | *Pisum sativum* | JBDLMJ000000000 | - | - | - |
|  | **SWRI132** | Iran | *Triticum aestivum* | CP077079 | - | - | - |
| *Pseudomonas atacamensis* | R-90876 | France | polluted soil | OZ336863 | - | - | - |
|  | B21-026 | Canada | agricultural soil | CP087187 | - | - | - |
|  | GZ-3 | China | *Populus sp.* | CP160044 | - | - | - |
|  | SM1 | United Arab Emirates | *Phoenix dactylifera* | CP070503 | - | - | - |
|  | **SWRI76** | Iran | *Triticum aestivum* | CP077081 | - | - | - |
| *Pseudomonas azotoformans* | **DSM 18862** | Japan | Oryza sativa | LT629702 | - | - | - |
|  | NRBC 12693 | Japan | *Oryza sativa* | BCZW01000000 | - | - | - |
| *Pseudomonas beijingensis* | FP2034 | China | plant rhizosphere | CP117451 | - | - | - |
|  | **FP830** | China | plant rhizosphere | CP117425 | - | - | - |
|  | FP2262 | China | plant rhizosphere | CP117443 | - | - | - |
| *Pseudomonas bijieensis* | **LMG 31948** | China | cornfiled soil | CP048810 | - | - | - |
| *Pseudomonas bohemica* | **LMG 30182** | Czech Republic | bark beetle | NKHL00000000 | - | - | - |
| *Pseudomonas brassicacearum* | **DSM 13227** | France | *Brassica napus* | JBHMAN000000000 | - | - | - |
| *Pseudomonas chlororaphis subs. chlororaphis* | **ATCC 9446** | - | plant roots | CP144767 | - | - | - |
|  | NCCB 47033 | - | - | CP118137 | - | - | - |
| *Pseudomonas chlororaphis subs. piscium* | ChPhzS135 | France | bulk soil | CP027738 | - | - | - |
|  | ChPhzS140e | France | bulk soil | CP027740 | - | - | - |
| *Pseudomonas crudilactis* | **UCMA 17988** | France | raw milk | WXVV00000000 | - | - | - |
| *Pseudomonas cyclaminis* | **MAFF 301449** | Japam | *Cyclamen* sp. | JADDUM010000000 | - | - | - |
| *Pseudomonas edaphica* | **LMG 30152** | Spain | *Cistus ladanifer* | VBVZ00000000 | - | - | - |
|  | PA-5-11F | UK | *Prunus avium* | WKCN00000000 | - | - | - |
| *Pseudomonas extremorientalis* | **LMG 19695** | Russia | drinking water reservoir | LT629708 | - | - | - |
| *Pseudomonas farris* | **SWRI79** | Iran | *Triticum aestivum* | JAHSTV000000000 | - | - | - |
| *Pseudomonas fluorescens* | **ATCC 13525** | UK | Pre-filter water-works tanks | LT907842 | - | - | - |
|  | **NBRC14160** | UK | Pre-filter water-works tanks | LHVP01000000 | - | - | - |
|  | **NCTC10038** | UK | Pre-filter water-works tanks | LS483372 | - | - | - |
|  | FP2327 | China | plant rhizosphere | CP117438 | - | - | - |
| *Pseudomonas folii* | **LMG 32142** | Canada | *Triticum aestivum* | JACONW000000000 | - | - | - |
| *Pseudomonas frederiksbergensis* | **DSM 13022** | Denmark | soil from a gasification site | FNTF00000000 | - | - | - |
|  | AS1 | South Korea | arsenic-contaminated soil | CP018319 | - | - | - |
| *Pseudomonas germanica* | **LMG 32353** | Spain | *Iris germanica* | CP071586 | - | - | - |
|  | P9-87 | Denmark | grassland soil | CP125372 | - | - | - |
|  | R-92842 | Austria | cyanide-polluted groundwater | OZ346140 | - | - | - |
| *Pseudomonas graminis* | 230d4 | Russia | - | - | PQ614800 | PQ628021 | PQ627991 |
|  | AM01 | China | - | - | PV355175 | PV366780 | PV366779 |
|  | UASWS1507 | Switzerland | *Malus* sp. | MDEN00000000 | - | - | - |
|  | **DSM 11363** | Germany | grasses | FOHW00000000 | - | - | - |
| *Pseudomonas granadensis* | **LMG 27940** | Spain | soil | LT629778 | - | - | - |
|  | PF29 | Italy | dairy product | - | MF838687 | MG022036 | MG021892 |
| *Pseudomonas grimontii* | **DSM 17515** | France | mineral water | FNKM00000000 | - | - | - |
|  | BIGb0428 | - | - | JANUER000000000 | - | - | - |
| *Pseudomonas hamedanensis* | **LMG 32041** | Iran | *Triticum aestivum* | CP077091 | - | - | - |
| *Pseudomonas iranensis* | **SWRI54** | Iran | *Triticum aestivum* | CP077092 | - | - | - |
| *Pseudomonas japonica* | **JCM 21532** | Japan | sewage treatment plant | BBIR00000000 | - | - | - |
| *Pseudomonas kilonensis* | **DSM 13647** | Germany | agricultural soil | LHVH00000000 | - | - | - |
|  | **DSM 16610** | South Korea | agricultural soil | JAAQYM000000000 | - | - | - |
| *Pseudomonas lactis* | **DSM 29167** | Germany | *raw milk* | JYLO01000000 | - | - | - |
| *Pseudomonas lurida* | **DSM 15835** | Belgium | *Triticum aestivum* | PDJB00000000 | - | - | - |
| *Pseudomonas lutea* | CFBP13723 | France | *Brassica napus* | JACYNP000000000 | - | - | - |
|  | **DSM 17257** | Spain | Rhizosphere of grasses | JRMB00000000 | - | - | - |
| *Pseudomonas marginalis* | **ATCC 10844** | USA | *Cichorium intybus* | VFEQ01000000 | - | - | - |
| *Pseudomonas moraviensis* | **LMG 24280** | Czech Republic | soil | LT629788 | - | - | - |
|  | R-92698 | Germany | polluted soil | OZ344932 | - | - | - |
|  | OTU5BARRA1 | France | *Arabidopsis thaliana* | CP099608 | - | - | - |
| *Pseudomonas moraviensis subsp. stanleyae* | **#71** | USA | *Stanleya pinnata* | JAADJI000000000 | - | - | - |
| *Pseudomonas paracarnis* | SAR266 | USA | *Homo sapiens* | JBQIHT010000000 | - | - | - |
|  | GTVSS-025 | USA | *Eruca sativa* | CP176709 | - | - | - |
|  | **LMG 31846** | Germany | dry aged refrigerated beef | JBQIHT010000000 | - | - | - |
| *Pseudomonas paralactis* | SWRI70 | Iran | *Triticum aestivum* | JABWQI000000000 | - | - | - |
|  | **DSM 29164** | Germany | raw milk | JYLN01000000 | - | - | - |
| *Pseudomonas pergaminensis* | **1008** | Argentina | *Triticum aestivum* | CP078013 | - | - | - |
| *Pseudomonas poae* | B12_1 | Denmark | *Triticum aestivum* | CP142193 | - | - | - |
|  | B12_4 | Denmark | *Triticum aestivum* | CP142150 | - | - | - |
|  | RE*1-1-14 | Austria | endorhiza sugar beet" | CP004045 | - | - | - |
|  | **DSM 14936** | Germany | phyllosphere of grasses | JYLI00000000 | - | - | - |
| *Pseudomonas putida* | **NBRC 14164** | - | soil | AP013070 | - | - | - |
|  | **NCTC 10936** | - | soil | CP101910 | - | - | - |
| *Pseudomonas rhizophila* | S211 | Tunisia | Rhizospheric soil | CP024081 | - | - | - |
|  | R104 | Netherlands | *Limonium vulgare* | CAXAOX000000000 | - | - | - |
|  | AS1 | Czech Republic | liquid enrichment culture | CP182239 | - | - | - |
|  | R-93403 | Netherlands | liquid enrichment culture | OZ346973 | - | - | - |
| *Pseudomonas salmasensis* | POE54 | Italy | *Solanum lycopersicum* | CP152477 | - | - | - |
|  | **SWRI126** | Iran | *Triticum aestivum* | CP077083 | - | - | - |
|  | LF044 | Canada | Commercially maple sap | JBRFIV010000000 | - | - | - |
| *Pseudomonas salomonii* | IPO3765 | Netherlands | *Agaricus bisporus* | JACAQV000000000 | - | - | - |
|  | **DSM 107228** | France | *Allium sativum* | MDFI01000000 | - | - | - |
|  | MAFF 212388 | Japan | *Allium sativum* | - | LC486820 | LC486835 | LC486850 |
| *Pseudomonas shirazensis* | R-92707 | Germany | polluted soil | OZ344942 | - | - | - |
|  | MPB05 | United Arab Emirates | *Citrullus colocynthis* | CP177040 | - | - | - |
|  | **LMG 32037** | Iran | *Triticum aestivum* | JABWRD000000000 | - | - | - |
|  | MPB13 | United Arab Emirates | *Citrullus colocynthis* | CP177047 | - | - | - |
| *Pseudomonas simiae* | WCS417 | Netherlands | *Triticum aestivum* | CP007637 | - | - | - |
|  | FP1649 | China | plant rhizosphere | CP117463 | - | - | - |
|  | FP55 | China | plant rhizosphere | CP117433 | - | - | - |
|  | **DSM 18861** | Spain | *Callithrix geoffroyi* | FOKB00000000 | - | - | - |
|  | FP1885 | China | plant rhizosphere | CP117456 | - | - | - |
| *Pseudomonas* sp*.* | IHBB745 | India | *Hippophae rhamnoides* | CP011507 | - | - | - |
|  | NCIMB 10586 | UK | soil | LR027557 | - | - | - |
|  | PF57 | Italy | dairy product | - | MF838676 | MG022025 | MG021881 |
|  | PF87 | Italy | dairy product | - | MF838656 | MG022005 | MG021861 |
|  | MFN1032 | - | *Homo sapiens* | - | KP119846 | KT350507 | KT350505 |
|  | SS101 | Netherlands | *Triticum aestivum* | AHPN00000000 | - | - | - |
|  | MTR3A | Tunisia | *Citrus tree* | - | LR214465 | LR214507 | LR214524 |
|  | MTR4 | Tunisia | *Citrus tree* | - | LR214466 | LR214508 | LR214525 |
|  | PF55 | Italy | dairy product | - | MF838641 | MG021990 | MG021846 |
|  | R1-4 | - | *Brassica napus* | - | KT358221 | KT358214 | KT358207 |
|  | BGCR2-9 | Belgium | *Solanum nigrum* | - | KT358222 | KT358215 | KT358208 |
| *Pseudomonas synxantha* | **DSM 18928** | USA | raw milk | JYLJ00000000 | - | - | - |
|  | R6-28-08 | USA | *Triticum aestivum* | CP027756 | - | - | - |
| *Pseudomonas tensinigenes* | **LMG 32032** | Belgium | *Triticum aestivum* | CP077089 | - | - | - |
| *Pseudomonas thivervalensis* | **LMG 21626** | France | *Brassica napus* | LHVE00000000 | - | - | - |
| *Pseudomonas tritici* | D277 | Antarctica | *Deschampsia antarctica* | JBJGXM000000000 | - | - | - |
|  | **SWRI145** | Iran | *Triticum aestivum* | CP077084 | - | - | - |
| *Pseudomonas triticicola* | **LMG 32052** | Iran | *Triticum aestivum* | JAHSTX000000000 | - | - | - |
| *Pseudomonas trivialis* | **DSM 14937** | Germany | phyllosphere of grasses | JYLK01000000 | - | - | - |
|  | W04-8 | Denmark | *Triticum aestivum* | CP142151 | - | - | - |
| *Pseudomonas viciae* | **GDMCC 1.1743** | China | *Vicia faba* | CP035088 | - | - | - |
| *Psychrobacillus psychrodurans* | LBUM564 | Canada | *Fragaria x ananassa* | CP189791 | - | - | - |
|  | **DSM 11713** | Egypt | garden soil | JAMKBK000000000 | - | - | - |
| *Psychrobacillus psychrotolerans* | **DSM 11706** | Germany | soil | FOXU00000000 | - | - | - |
| *Solibacillus isronensis* | **B3W22** | India | upper atmosphere | AMCK00000000 | - | - | - |
| *Solibacillus kalamii* | **ISSFR-015** | USA | HEPA filter from International Space Station | NHNT00000000 | - | - | - |
| *Solibacillus silvestris* | SAF-065 | USA | clean room floor | JBQCJW000000000 | - | - | - |
|  | StLB046 | Japan | *Solanum tuberosum* | AP012157 | - | - | - |
| *Terribacillus goriensis* | MP602 | China | *Cryptomeria fortunei* | CP008876 | - | - | - |
| *Terribacillus halophilus* | **DSM 21620** | Japan | field soil | FMZB00000000 | - | - | - |
| *Terribacillus saccharophilus* | DSM 18252 | South Korea | coastal water | NJAQ00000000 | - | - | - |
|  | **DSM 21619** | Japan | field soil | FOCD00000000 | - | - | - |

**Table S3.** List of bacterial strains from the families *Bacillaceae*. *Paenibacillaceae* and *Pseudomonadaceae* isolated from different plant compartments organs of *Triticum aestivum* from harvested in different fields in Spain (SP) and The Netherlands (NL). The newly sequences generated in this study are in italics.

| **Strain** | **Species** | **Location** | **Agricultural practice / soil type** | **Isolation source** | **GenBank Accession** | | |
| --- | --- | --- | --- | --- | --- | --- | --- |
|  |  |  |  |  | 16S rRNA | *gyrB* | *rpoD* |
| **B29** | *Psychrobacillus psychrodurans* | SP | Conventional irrigated | Root | *PX769958* | *PX781625* | *-* |
| **B30** | *Phytopseudomonas prunonensis* | SP | Conventional irrigated | Root | *PX769969* | *PX781636* | *PX781680* |
| **B34** | *Pseudomonas beijingensis* | SP | Conventional irrigated | Root | *PX769999* | *PX781666* | *PX781710* |
| **B37** | *Pseudomonas* sp. | SP | Conventional irrigated | Leaf | *PX769984* | *PX781651* | *PX781695* |
| **B41** | *Pseudomonas* sp*.* | SP | Conventional irrigated | Leaf | *PX769960* | *PX781627* | *PX781671* |
| **B44** | *Pseudomonas poae* | SP | Conventional irrigated | Leaf | *PX769977* | *PX781644* | *PX781688* |
| **B76** | *Paenibacillus amylolyticus* | SP | Conventional irrigated | Root | *PX769940* | *PX781608* | *-* |
| **B77** | *Pseudomonas chlororaphis subsp. chloroaphis* | SP | Conventional irrigated | Root | *PX769998* | *PX781665* | *PX781709* |
| **B79** | *Pseudomonas chlororaphis subsp. chloroaphis* | SP | Conventional irrigated | Root | *PX769997* | *PX781664* | *PX781708* |
| **B93** | *Paenibacillus nuruki* | SP | Conventional irrigated | Grain | *PX769947* | *PX781615* | *-* |
| **B110** | *Pseudomonas simiae* | SP | Conventional irrigated | Root | *PX769979* | *PX781646* | *PX781690* |
| **B119** | *Pseudomonas rhizophila* | SP | Conventional irrigated | Root | *PX770001* | *PX781668* | *PX781712* |
| **B126** | *Paenibacillus amylolyticus* | SP | Conventional irrigated | Leaf | *PX769941* | *PX781609* | *-* |
| **B136** | *Pseudomonas* sp*.* | SP | Conventional irrigated | Grain | *PX769992* | *PX781659* | *PX781703* |
| **B153** | *Pseudomonas* sp*.* | SP | Organic non-irrigated | Root | *PX770000* | *PX781667* | *PX781711* |
| **B155** | *Pseudomonas* sp*.* | SP | Organic non-irrigated | Root | *PX769962* | *PX781629* | *PX781673* |
| **B160** | *Pseudomonas* sp*.* | SP | Organic non-irrigated | Root | *PX769961* | *PX781628* | *PX781672* |
| **B164** | *Pseudomonas paracarnis* | SP | Organic non-irrigated | Leaf | *PX769986* | *PX781653* | *PX781697* |
| **B165** | *Pseudomonas* sp*.* | SP | Organic non-irrigated | Leaf | *PX769993* | *PX781660* | *PX781704* |
| **B192** | *Terribacillus saccharophilus* | SP | Organic non-irrigated | Root | *PX769956* | *PX781603* | *-* |
| **B194** | *Paenibacillus amylolyticus* | SP | Organic non-irrigated | Root | *PX769943* | *PX781611* | *-* |
| **B198** | *Peribacillus frigorotolerans* | SP | Organic non-irrigated | Root | *PX769955* | *PX781623* | *-* |
| **B201** | *Paenibacillus nuruki* | SP | Organic non-irrigated | Leaf | *PX769946* | *PX781614* | *-* |
| **B210** | *Pseudomonas* sp*.* | SP | Organic non-irrigated | Leaf | *PX769989* | *PX781656* | *PX781700* |
| **B216** | *Peribacillus frigorotolerans* | SP | Organic non-irrigated | Root | *PX769954* | *PX781622* | *-* |
| **B221** | *Bacillus pumilus* | SP | Organic non-irrigated | Root | *PX769953* | *PX781621* | *-* |
| **B253** | *Pseudomonas* sp*.* | SP | Conventional non-irrigated | Root | *PX769965* | *PX781632* | *PX781676* |
| **B254** | *Pseudomonas tritici* | SP | Conventional non-irrigated | Root | *PX769981* | *PX781648* | *PX781692* |
| **B266** | *Pseudomonas paracarnis* | SP | Conventional non-irrigated | Grain | *PX769988* | *PX781655* | *PX781699* |
| **B287** | *Paenibacillus amylolyticus* | SP | Conventional non-irrigated | Root | *PX769937* | *PX781605* | *-* |
| **B293** | *Bacillus velezensis* | SP | Conventional non-irrigated | Leaf | *PX769948* | *PX781616* | *-* |
| **B298** | *Pseudomonas sp.* | SP | Conventional non-irrigated | Leaf | *PX769968* | *PX781635* | *PX781679* |
| **B304** | *Pseudomonas lactis* | SP | Conventional non-irrigated | Grain | *PX769990* | *PX781657* | *PX781701* |
| **B338** | *Paenibacillus amylolyticus* | SP | Conventional non-irrigated | Root | *PX769938* | *PX781606* | *-* |
| **B342** | *Paenibacillus pabuli* | SP | Conventional non-irrigated | Root | *PX769944* | *PX781612* | *-* |
| **B353** | *Bacillus velezensis* | SP | Conventional non-irrigated | Leaf | *PX769949* | *PX781617* | *-* |
| **B362** | *Pseudomonas atacamensis* | SP | Conventional non-irrigated | Grain | *PX769959* | *PX781626* | *PX781670* |
| **B367** | *Pseudomonas fluorescens* | SP | Conventional non-irrigated | Grain | *PX769985* | *PX781652* | *PX781696* |
| **B393** | *Bacillus pumilus* | NL | Light/medium clay | roots | *PX769952* | *PX781620* | *-* |
| **B411** | *Pseudomonas* sp*.* | NL | Light/medium clay | Grain | *PX769987* | *PX781654* | *PX781698* |
| **B437** | *Solibacillus* sp. | NL | Light/medium clay | roots | *PX769957* | *PX781624* | *-* |
| **B446** | *Pseudomonas poae* | NL | Light/medium clay | Root | *PX769972* | *PX781639* | *PX781683* |
| **B451** | *Pseudomonas* sp. | NL | Light/medium clay | Leaf | *PX769991* | *PX781658* | *PX781702* |
| **B452** | *Pseudomonas paralactis* | NL | Light/medium clay | Leaf | *PX769994* | *PX781661* | *PX781705* |
| **B484** | *Pseudomonas tensinigenes* | NL | Light/medium clay | Root | *PX769964* | *PX781631* | *PX781675* |
| **B487** | *Bacillus licheniformis* | NL | Light/medium clay | roots | *PX769951* | *PX781619* | *-* |
| **B497** | *Pseudomonas cyclaminis* | NL | Light/medium clay | Leaf | *PX769973* | *PX781640* | *PX781684* |
| **B503** | *Pseudomonas poae* | NL | Light/medium clay | Grain | *PX769974* | *PX781641* | *PX781685* |
| **B505** | *Pseudomonas shirazensis* | NL | Light/medium clay | Grain | *PX769971* | *PX781638* | *PX781682* |
| **B527** | *Paenibacillus amylolyticus* | NL | Light/medium clay | roots | *PX769939* | *PX781607* | *-* |
| **B532** | *Pseudomonas* sp. | NL | Light/medium clay | Root | *PX769982* | *PX781649* | *PX781693* |
| **B538** | *Pseudomonas simiae* | NL | Light/medium clay | Root | *PX769980* | *PX781647* | *PX781691* |
| **B539** | *Phytopseudomonas seleniipraecipitans* | NL | Light/medium clay | Root | *PX769970* | *PX781637* | *PX781681* |
| **B546** | *Pseudomonas* sp. | NL | Light/medium clay | Grain | *PX769995* | *PX781662* | *PX781706* |
| **B571** | *Pseudomonas* sp. | NL | Light/medium clay | Root | *PX769966* | *PX781633* | *PX781677* |
| **B573** | *Pseudomonas asgharzadehiana* | NL | Light/medium clay | Root | *PX769983* | *PX781650* | *PX781694* |
| **B577** | *Pseudomonas frederiksbergensis* | NL | Light/medium clay | Root | *PX769996* | *PX781663* | *PX781707* |
| **B583** | *Pseudomonas poae* | NL | Light/medium clay | Root | *PX769976* | *PX781643* | *PX781687* |
| **B588** | *Pseudomonas simiae* | NL | Light/medium clay | Leaf | *PX769978* | *PX781645* | *PX781689* |
| **B597** | *Pseudomonas graminis* | NL | Heavy clay | Leaf | *PX769967* | *PX781634* | *PX781678* |
| **B598** | *Paenibacillus amylolyticus* | NL | Heavy clay | Leaf | *PX769936* | *PX781604* | *-* |
| **B600** | *Pseudomonas poae* | NL | Heavy clay | Leaf | *PX769975* | *PX781642* | *PX781686* |
| **B602** | *Paenibacillus amylolyticus* | NL | Heavy clay | Leaf | *PX769942* | *PX781610* | *-* |
| **B604** | *Bacillus licheniformis* | NL | Heavy clay | Leaf | *PX769950* | *PX781618* | *-* |
| **B605** | *Pseudomonas germanica* | NL | Heavy clay | Leaf | *PX769963* | *PX781630* | *PX781674* |
| **B641** | *Paenibacillus nuruki* | NL | Very heavy clay | Leaf | *PX769945* | *PX781613* | *-* |

**Table S4.** Functional characterization of the *Pseudomonadaceae*. *Paenibacillaceae* and *Bacillaceae* isolates. following a multi-trait biochemical analysis of indole-3-acetic acid (IAA). ACC deaminase. nitrogen fixation (NF). urease activity. phosphorus solubilization index (PSI). siderophores production and bacterial motility. The values of PSI and IAA represent the mean ± standard deviation (n=3). while the signs “–” and “+” for NF and urease indicate negative or positive nitrogen fixation capacity urea degradation. respectively (n = 3). Empty cells stand for isolates not tested.

| Strain | Molecular Identification | IAA (µmol mL^-1^) | ACC deaminase (µmol mL^-1^) | NF | Urease | PSI | Siderophore  (%) | Motility  (cm) |
| --- | --- | --- | --- | --- | --- | --- | --- | --- |
| B29 | *Psychrobacillus psychrodurans* | 9.99 ± 0.27 | 34.52 ± 0.02 | - | - | 0 | 35.70 ± 2.48 | 42.00 ± 8.45 |
| B30 | *Phytopseudomonas prunonensis* | 40.23 ± 1.53 |  | +++ |  | 1.25 ± 0.01 |  |  |
| B34 | *Pseudomonas beijingensis* | 99.23 ± 8.39 |  | ++ |  | 1.26 ± 0.03 |  |  |
| B37 | *Pseudomonas* sp*.* | 76.90 ± 9.54 |  | - |  | 1.19 ± 0.01 |  |  |
| B41 | *Pseudomonas* sp*.* | 44.90 ± 3.00 | 40.90 ± 0.61 | +++ | ++ | 2.31 ± 0.10 | 18.64 ± 1.06 | 45.26 ± 1.37 |
| B44 | *Pseudomonas poae* | 0.06 ± 0.00 |  | ++ |  | 1.73 ± 0.14 |  |  |
| B76 | *Paenibacillus amylolyticus* | 9.57 ± 0.76 | 37.07 ± 1.04 | - | - | 0 | 38.32 ± 2.10 | 89.99 ± 0.01 |
| B77 | *Pseudomonas chlororaphis subs. chloroaphis* | 21.23 ± 0.58 |  | ++ |  | 0 |  |  |
| B79 | *Pseudomonas chlororaphis subs. chloroaphis* | 13.23 ± 0.58 |  | +++ |  | 0 |  |  |
| B93 | *Paenibacillus nuruki* | 11.36 ± 0.57 | 33.60 ± 0.80 | + | - | 1.09 ± 0.01 | 29.56 ± 6.96 | 90.00 ± 0.00 |
| B110 | *Pseudomonas simiae* | 59.56 ± 3.79 | 38.44 ± 1.29 | +++ | - | 2.28 ± 0.16 | 41.70 ± 5.34 | 40.85 ± 1.66 |
| B119 | *Pseudomonas rhizophila* | 59.56 ± 5.69 |  | +++ |  | 1.61 ± 0.18 |  |  |
| B126 | *Paenibacillus amylolyticus* | 2.31 ± 0.29 | 34.24 ± 0.98 | - | - | 0 | 27.27 ± 2.10 | 47.95 ± 16.20 |
| B136 | *Pseudomonas* sp*.* | 37.56 ± 2.51 |  | ++ |  | 1.21 ± 0.03 |  |  |
| B153 | *Pseudomonas* sp*.* | 38.56 ± 2.31 |  | +++ |  | 2.02 ± 0.14 |  |  |
| B155 | *Pseudomonas* sp*.* | 0.36 ± 0.27 |  | +++ |  | 2.23 ± 0.05 |  |  |
| B160 | *Pseudomonas* sp*.* | 38.23 ± 2.89 |  | +++ |  | 2.03 ± 0.37 |  |  |
| B164 | *Pseudomonas paracarnis* | 48.56 ± 6.66 |  | ++ |  | 1.38 ± 0.06 |  |  |
| B165 | *Pseudomonas* sp*.* | 38.56 ± 3.51 |  | + |  | 1.21 ± 0.03 |  |  |
| B192 | *Terribacillus saccharophilus* | 13.69 ± 0.32 | 37.00 ± 2.94 | - | + | 0 | 37.83 ± 4.13 | 14.48 ± 4.54 |
| B194 | *Paenibacillus amylolyticus* | 2.66 ± 0.14 | 35.76 ± 1.83 | - | - | 0 | 1.05 ± 0.35 | 90.00 ± 0.00 |
| B198 | *Peribacillus frigorotolerans* | 5.41 ± 0.24 | 37.60 ± 1.64 | - | + | 0 | 29.37 ± 3.05 | 36.75 ± 14.7 |
| B201 | *Paenibacillus nuruki* | 11.90 ± 0.34 | 34.62 ± 0.75 | - | + | 1.08 ± 0.03 | 5.87 ± 0.42 | 90.00 ± 0.00 |
| B210 | *Pseudomonas* sp*.* | 30.90 ± 1.00 |  | ++ |  | 1.15 ± 0.04 |  |  |
| B216 | *Peribacillus frigorotolerans* | 0.78 ± 0.14 | 35.68 ± 1.07 | + | - | 1.10 ± 0.00 | 40.98 ± 9.46 | 20.20 ± 5.72 |
| B221 | *Bacillus pumilus* | 1.81 ± 0.58 | 39.35 ± 0.78 | - | + | 0 | 0.93 ± 0.40 | 90.00 ± 0.00 |
| B253 | *Pseudomonas* sp*.* | 28.90 ± 1.73 |  | + |  | 0 |  |  |
| B254 | *Pseudomonas tritici* | 109.6 ± 3.06 | 38.32 ± 0.53 | ++ | + | 2.84 ± 0.23 | 21.44 ± 3.85 | 15.84 ± 0.13 |
| B266 | *Pseudomonas paracarnis* | 28.23 ± 2.08 |  | ++ |  | 0 |  |  |
| B287 | *Paenibacillus amylolyticus* | 19.56 ± 1.24 | 43.53 ± 2.30 | + | - | 0 | 28.32 ± 0.35 | 90.00 ± 0.00 |
| B293 | *Bacillus velezensis* | 4.06 ± 0.09 | 45.70 ± 3.12 | - | - | 0 | 31.12 ± 6.99 | 90.00 ± 0.00 |
| B298 | *Pseudomonas* sp*.* | 29.23 ± 2.08 |  | +++ |  | 1.96 ± 0.15 |  |  |
| B304 | *Pseudomonas lactis* | 26.56 ± 1.53 |  | - |  | 1.13 ± 0.02 |  |  |
| B338 | *Paenibacillus amylolyticus* | 28.17 ± 5.32 | 36.97 ± 0.41 | - | - | 0 | 1.17 ± 0.40 | 90.00 ± 0.00 |
| B342 | *Paenibacillus pabuli* | 73.30 ± 5.66 | 38.08 ± 1.03 | + | - | 0 | 28.64 ± 5.56 | 90.00 ± 0.00 |
| B353 | *Bacillus velezensis* | 4.78 ± 0.18 | 47.26 ± 6.13 | - | + | 1.29 ± 0.03 | 39.37 ± 7.34 | 90.00 ± 0.00 |
| B362 | *Pseudomonas iranensis* | 51.23 ± 5.03 | 40.13 ± 0.40 | +++ | + | 2.36 ± 0.08 | 35.17 ± 4.42 | 44.50 ± 1.75 |
| B367 | *Pseudomonas flourescens* | 20.56 ± 2.89 | 37.98 ± 0.73 | +++ | ++ | 2.95 ± 0.29 | 38.18 ± 4.72 | 43.64 ± 0.23 |
| B393 | *Bacillus pumilus* | 2.81 ± 0.16 | 48.77 ± 1.67 | - | - | 1.30 ± 0.02 | 2.57 ± 0.40 | 90.00 ± 0.00 |
| B411 | *Pseudomonas* sp*.* | 75.56 ± 7.02 | 38.70 ± 0.34 | +++ | ++ | 3.12 ± 0.11 | 36.57 ± 1.40 | 39.91 ± 4.62 |
| B437 | *Solibacillus* sp*.* | 2.96 ± 0.14 | 35.02 ± 1.12 | - | - | 0 | 1.05 ± 0.35 | 35.55 ± 2.59 |
| B446 | *Pseudomonas poae* | 57.90 ± 3.46 |  | ++ |  | 1.31 ± 0.05 |  |  |
| B451 | *Pseudomonas* sp*.* | 66.23 ± 7.51 | 37.90 ± 0.19 | +++ | ++ | 1.91 ± 0.50 | 35.31 ± 4.11 | 87.41 ± 0.06 |
| B452 | *Pseudomonas paralactis* | 31.90 ± 3.00 |  | ++ |  | 1.24 ± 0.18 |  |  |
| B484 | *Pseudomonas tensinigenes* | 77.23 ± 4.62 |  | +++ |  | 1.35 ± 0.14 |  |  |
| B487 | *Bacillus licheniformis* | 3.35 ± 0.16 | 40.06 ± 0.78 | - | - | 0 | 48.99 ± 0.60 | 90.00 ± 0.00 |
| B497 | *Pseudomonas cyclaminis* | 11.90 ± 1.00 |  | ++ |  | 0 |  |  |
| B503 | *Pseudomonas poae* | 34.56 ± 1.53 |  | ++ |  | 1.63 ± 0.18 |  |  |
| B505 | *Pseudomonas shirazensis* | 114.6 ± 6.66 |  | ++ |  | 0 |  |  |
| B527 | *Paenibacillus amylolyticus* | 7.06 ± 0.26 | 37.37 ± 0.45 | - | - | 0 | 23.43 ± 3.15 | 90.00 ± 0.00 |
| B532 | *Pseudomonas* sp*.* | 16.23 ± 1.53 |  | +++ |  | 1.95 ± 0.09 |  |  |
| B538 | *Pseudomonas simiae* | 53.90 ± 6.00 |  | +++ |  | 1.78 ± 0.06 |  |  |
| B539 | *Phytopseudomonas seleniipraecipitans* | 50.56 ± 6.66 |  | - |  | 0 |  |  |
| B546 | *Pseudomonas* sp. | 46.56 ± 1.16 |  | +++ |  | 1.79 ± 0.13 |  |  |
| B571 | *Pseudomonas* sp. | 79.56 ± 7.02 |  | ++ |  | 1.40 ± 0.09 |  |  |
| B573 | *Pseudomonas asgharzadehiana* | 143.6 ± 19.5 | 40.38 ± 0.28 | ++ | ++ | 2.96 ± 0.11 | 45.15 ± 8.16 | 23.80 ± 2.57 |
| B577 | *Pseudomonas frederiksbergensis* | 32.23 ± 3.79 |  | +++ |  | 1.60 ± 0.06 |  |  |
| B583 | *Pseudomonas poae* | 75.56 ± 10.3 |  | +++ |  | 1.78 ± 0.15 |  |  |
| B588 | *Pseudomonas simiae* | 50.23 ± 4.51 | 38.71 ± 0.36 | +++ | ++ | 3.21 ± 0.10 | 32.87 ± 4.97 | 44.13 ± 5.26 |
| B597 | *Pseudomonas graminis* | 38.90 ± 4.00 |  | +++ |  | 1.77 ± 0.10 |  |  |
| B598 | *Paenibacillus amylolyticus* | 6.97 ± 0.72 | 35.63 ± 1.66 | - | + | 0 | 2.45 ± 0.35 | 90.00 ± 0.00 |
| B600 | *Pseudomonas poae* | 52.90 ± 2.65 |  | - |  | 1.80 ± 0.05 |  |  |
| B602 | *Paenibacillus amylolyticus* | 19.74 ± 0.53 | 37.09 ± 1.31 | - | - | 0 | 25.17 ± 4.90 | 90.00 ± 0.00 |
| B604 | *Bacillus licheniformis* | 2.96 ± 0.23 | 34.26 ± 1.01 | + | - | 0 | 40.26 ± 1.84 | 90.00 ± 0.00 |
| B605 | *Pseudomonas germanica* | 65.23 ± 8.08 |  | + |  | 0 |  |  |
| B641 | *Paenibacillus nuruki* | 11.72 ± 0.14 | 36.07 ± 1.33 | + | - | 1.28 ± 0.03 | 32.40 ± 5.84 | 90.00 ± 0.00 |

**Table S5.** Row-normalized values of the plant growth-promoting traits used to calculate the composite performance score for bacterial strain selection. Values were normalized independently for each trait to a 0–1 scale, where 0 and 1 correspond to the lowest and highest observed values, respectively, within each bacterial family. Equal weighting was assigned to all traits when calculating the composite performance score. Dashes (–) indicate traits that were not evaluated for *Pseudomonadaceae* strains. Abbreviations: PSI, phosphate solubilization index; IAA, Salkowski-reactive indolic compounds; NF, presumptive nitrogen-fixing potential (growth on nitrogen-free medium); Salinity, tolerance to saline stress; ACC, ACC deaminase activity.

| Family | Strain | PSI | IAA | NF | Salinity | Siderophores | ACC | Urease | Agroecosystem |
| --- | --- | --- | --- | --- | --- | --- | --- | --- | --- |
| *Bacillaceae/Paenibacillaceae* | B29 | 0.00 | 0.13 | 0.00 | 0.35 | 0.72 | 0.06 | 0.00 | Mediterranean |
|  | B76 | 0.00 | 0.12 | 0.00 | 0.11 | 0.78 | 0.23 | 0.00 | Mediterranean |
|  | B93 | 0.85 | 0.15 | 1.00 | 0.05 | 0.60 | 0.00 | 0.00 | Mediterranean |
|  | B126 | 0.00 | 0.02 | 0.00 | 0.04 | 0.55 | 0.04 | 0.00 | Mediterranean |
|  | B192 | 0.00 | 0.18 | 0.33 | 0.19 | 0.77 | 0.22 | 1.00 | Mediterranean |
|  | B194 | 0.00 | 0.03 | 0.33 | 0.03 | 0.00 | 0.14 | 0.00 | Mediterranean |
|  | B198 | 0.00 | 0.06 | 0.00 | 0.16 | 0.59 | 0.26 | 0.00 | Mediterranean |
|  | B201 | 0.85 | 0.15 | 1.00 | 0.00 | 0.10 | 0.07 | 0.00 | Mediterranean |
|  | B216 | 0.85 | 0.00 | 1.00 | 0.21 | 0.83 | 0.14 | 1.00 | Mediterranean |
|  | B221 | 0.00 | 0.01 | 0.00 | 1.00 | 0.00 | 0.38 | 0.67 | Mediterranean |
|  | B287 | 0.00 | 0.26 | 1.00 | 0.41 | 0.57 | 0.65 | 0.00 | Mediterranean |
|  | B293 | 0.00 | 0.05 | 0.67 | 0.81 | 0.63 | 0.80 | 0.33 | Mediterranean |
|  | B338 | 0.00 | 0.38 | 0.33 | 0.30 | 0.01 | 0.22 | 0.67 | Mediterranean |
|  | B342 | 0.00 | 1.00 | 1.00 | 0.16 | 0.58 | 0.30 | 0.00 | Mediterranean |
|  | B353 | 1.00 | 0.06 | 0.67 | 0.79 | 0.80 | 0.90 | 0.00 | Mediterranean |
|  | B393 | 1.00 | 0.03 | 0.33 | 0.83 | 0.03 | 1.00 | 1.00 | Atlantic |
|  | B437 | 0.00 | 0.03 | 0.00 | 0.05 | 0.00 | 0.09 | 0.33 | Atlantic |
|  | B487 | 0.00 | 0.04 | 0.67 | 0.75 | 1.00 | 0.43 | 0.00 | Atlantic |
|  | B527 | 0.00 | 0.09 | 0.33 | 0.06 | 0.47 | 0.25 | 0.00 | Atlantic |
|  | B598 | 0.00 | 0.09 | 0.33 | 0.25 | 0.03 | 0.13 | 0.33 | Atlantic |
|  | B602 | 0.00 | 0.26 | 0.67 | 0.17 | 0.50 | 0.23 | 0.33 | Atlantic |
|  | B604 | 0.00 | 0.03 | 1.00 | 0.68 | 0.82 | 0.04 | 0.33 | Atlantic |
|  | B641 | 1.00 | 0.15 | 1.00 | 0.07 | 0.66 | 0.16 | 0.00 | Atlantic |
| *Pseudomonadaceae* | B30 | 0.39 | 0.28 | 1.00 | - | - | - | - | Mediterranean |
|  | B34 | 0.39 | 0.69 | 0.67 | - | - | - | - | Mediterranean |
|  | B37 | 0.37 | 0.54 | 0.00 | - | - | - | - | Mediterranean |
|  | B41 | 0.72 | 0.31 | 1.00 | - | - | - | - | Mediterranean |
|  | B44 | 0.54 | 0.00 | 0.67 | - | - | - | - | Mediterranean |
|  | B77 | 0.00 | 0.15 | 0.67 | - | - | - | - | Mediterranean |
|  | B79 | 0.00 | 0.09 | 1.00 | - | - | - | - | Mediterranean |
|  | B110 | 0.71 | 0.41 | 1.00 | - | - | - | - | Mediterranean |
|  | B119 | 0.50 | 0.41 | 1.00 | - | - | - | - | Mediterranean |
|  | B136 | 0.38 | 0.26 | 0.67 | - | - | - | - | Mediterranean |
|  | B153 | 0.63 | 0.27 | 1.00 | - | - | - | - | Mediterranean |
|  | B155 | 0.69 | 0.00 | 1.00 | - | - | - | - | Mediterranean |
|  | B160 | 0.63 | 0.27 | 1.00 | - | - | - | - | Mediterranean |
|  | B164 | 0.43 | 0.34 | 0.67 | - | - | - | - | Mediterranean |
|  | B165 | 0.38 | 0.27 | 0.33 | - | - | - | - | Mediterranean |
|  | B210 | 0.36 | 0.21 | 0.67 | - | - | - | - | Mediterranean |
|  | B253 | 0.00 | 0.20 | 0.33 | - | - | - | - | Mediterranean |
|  | B254 | 0.88 | 0.76 | 0.67 | - | - | - | - | Mediterranean |
|  | B266 | 0.00 | 0.20 | 0.67 | - | - | - | - | Mediterranean |
|  | B298 | 0.61 | 0.20 | 1.00 | - | - | - | - | Mediterranean |
|  | B304 | 0.35 | 0.18 | 0.00 | - | - | - | - | Mediterranean |
|  | B362 | 0.74 | 0.36 | 1.00 | - | - | - | - | Mediterranean |
|  | B367 | 0.92 | 0.14 | 1.00 | - | - | - | - | Mediterranean |
|  | B411 | 0.97 | 0.53 | 1.00 | - | - | - | - | Atlantic |
|  | B446 | 0.41 | 0.40 | 0.67 | - | - | - | - | Atlantic |
|  | B451 | 0.60 | 0.46 | 1.00 | - | - | - | - | Atlantic |
|  | B452 | 0.39 | 0.22 | 0.67 | - | - | - | - | Atlantic |
|  | B484 | 0.42 | 0.54 | 1.00 | - | - | - | - | Atlantic |
|  | B497 | 0.00 | 0.08 | 0.67 | - | - | - | - | Atlantic |
|  | B503 | 0.51 | 0.24 | 0.67 | - | - | - | - | Atlantic |
|  | B505 | 0.00 | 0.80 | 0.67 | - | - | - | - | Atlantic |
|  | B532 | 0.61 | 0.11 | 1.00 | - | - | - | - | Atlantic |
|  | B538 | 0.55 | 0.38 | 1.00 | - | - | - | - | Atlantic |
|  | B539 | 0.00 | 0.35 | 0.00 | - | - | - | - | Atlantic |
|  | B546 | 0.56 | 0.32 | 1.00 | - | - | - | - | Atlantic |
|  | B571 | 0.44 | 0.55 | 0.67 | - | - | - | - | Atlantic |
|  | B573 | 0.92 | 1.00 | 0.67 | - | - | - | - | Atlantic |
|  | B577 | 0.50 | 0.22 | 1.00 | - | - | - | - | Atlantic |
|  | B583 | 0.55 | 0.53 | 1.00 | - | - | - | - | Atlantic |
|  | B588 | 1.00 | 0.35 | 1.00 | - | - | - | - | Atlantic |
|  | B597 | 0.55 | 0.27 | 1.00 | - | - | - | - | Atlantic |
|  | B600 | 0.56 | 0.37 | 0.00 | - | - | - | - | Atlantic |
|  | B605 | 0.00 | 0.45 | 0.33 | - | - | - | - | Atlantic |

**Table S6**. Tolerance of *Pseudomonadaceae*. *Bacillaceae* and *Paenibacillaceae* to pH variation. Data are presented as means of absorbance (OD_600_) ± standard deviation (n = 3). Different letters indicate significant differences among pH levels within the same isolate, according to Tukey’s test at 5% significance.

| Strain | Species | pH variation | | | |  |
| --- | --- | --- | --- | --- | --- | --- |
|  |  | **pH 4** | **pH 5** | **pH 7** | **pH 8** | ***Fprob*** |
| B29 | *Psychrobacillus psychrodurans* | 0.220 ± 0.011ᵇ | 0.273 ± 0.083ᵇ | 0.496 ± 0.025ᵃ | 0.556 ± 0.102ᵃ | **<0.001** |
| B41 | *Pseudomonas* sp. | 0.270 ± 0.029ᵇ | 0.596 ± 0.009ᵃ | 0.649 ± 0.010ᵃ | 0.649 ± 0.056ᵃ | **<0.001** |
| B76 | *Paenibacillus amylolyticus* | 0.297 ± 0.001ᵇ | 0.310 ± 0.003ᵇ | 0.558 ± 0.049ᵃ | 0.641 ± 0.043ᵃ | **<0.001** |
| B93 | *Paenibacillus nuruki* | 0.189 ± 0.009ᵇ | 0.724 ± 0.043ᵃ | 0.461 ± 0.269ᵃᵇ | 0.722 ± 0.093ᵃ | **<0.006** |
| B110 | *Pseudomonas simiae* | 0.327 ± 0.045ᶜ | 0.903 ± 0.003ᵃ | 0.852 ± 0.016ᵃᵇ | 0.825 ± 0.032ᵇ | **<0.001** |
| B126 | *Paenibacillus amylolyticus* | 0.231 ± 0.002ᶜ | 0.261 ± 0.002ᶜ | 0.705 ± 0.102ᵃ | 0.560 ± 0.012ᵇ | **<0.001** |
| B192 | *Terribacillus saccharophilus* | 0.243 ± 0.003ᵇ | 0.962 ± 0.004ᵃ | 0.947 ± 0.029ᵃ | 1.062 ± 0.159ᵃ | **<0.001** |
| B194 | *Paenibacillus amylolyticus* | 0.265 ± 0.164ᵇ | 0.305 ± 0.012ᵇ | 0.722 ± 0.001ᵃ | 0.726 ± 0.060ᵃ | **<0.001** |
| B198 | *Peribacillus frigorotolerans* | 0.404 ± 0.167ᵇ | 0.271 ± 0.086ᵇ | 0.793 ± 0.075ᵃ | 0.283 ± 0.007ᵇ | **<0.001** |
| B201 | *Paenibacillus nuruki* | 0.188 ± 0.007ᵇ | 0.798 ± 0.050ᵃ | 0.730 ± 0.005ᵃ | 0.772 ± 0.021ᵃ | **<0.001** |
| B216 | *Peribacillus frigorotolerans* | 0.252 ± 0.015ᵇ | 0.222 ± 0.018ᵇ | 0.558 ± 0.088ᵃ | 0.303 ± 0.010ᵇ | **<0.001** |
| B221 | *Bacillus pumilus* | 0.221 ± 0.001ᵃ | 0.224 ± 0.009ᵃ | 0.482 ± 0.261ᵃ | 0.413 ± 0.022ᵃ | 0.089 |
| B254 | *Pseudomonas tritici* | 0.168 ± 0.029ᵇ | 0.539 ± 0.027ᵃ | 0.576 ± 0.049ᵃ | 0.519 ± 0.012ᵃ | **<0.001** |
| B287 | *Paenibacillus amylolyticus* | 0.249 ± 0.003ᶜ | 0.303 ± 0.010ᶜ | 0.947 ± 0.173ᵃ | 0.632 ± 0.025ᵇ | **<0.001** |
| B293 | *Bacillus velezensis* | 0.456 ± 0.004ᵃ | 0.413 ± 0.022ᵃ | 0.497 ± 0.128ᵃ | 0.409 ± 0.015ᵃ | 0.347 |
| B338 | *Paenibacillus amylolyticus* | 0.234 ± 0.001ᶜ | 0.496 ± 0.029ᵇ | 0.842 ± 0.064ᵃ | 0.884 ± 0.073ᵃ | **<0.001** |
| B342 | *Paenibacillus pabuli* | 0.238 ± 0.007ᵇ | 0.400 ± 0.029ᵇ | 0.967 ± 0.053ᵃ | 1.008 ± 0.113ᵃ | **<0.001** |
| B353 | *Bacillus velezensis* | 0.509 ± 0.060ᵇ | 0.621 ± 0.051ᵃᵇ | 0.719 ± 0.044ᵃ | 0.664 ± 0.014ᵃ | **0.003** |
| B362 | *Pseudomonas iranensis* | 0.183 ± 0.021ᶜ | 0.558 ± 0.035ᵃ | 0.556 ± 0.022ᵃ | 0.483 ± 0.006ᵇ | **<0.001** |
| B367 | *Pseudomonas flourescens* | 0.273 ± 0.043ᵇ | 0.871 ± 0.004ᵃ | 0.871 ± 0.014ᵃ | 0.845 ± 0.016ᵃ | **<0.001** |
| B393 | *Bacillus pumilus* | 0.253 ± 0.015ᶜ | 0.793 ± 0.029ᵇ | 0.891 ± 0.042ᵃ | 0.862 ± 0.029ᵃᵇ | **<0.001** |
| B411 | *Pseudomonas* sp. | 0.258 ± 0.038ᵇ | 0.750 ± 0.010ᵃ | 0.730 ± 0.018ᵃ | 0.733 ± 0.007ᵃ | **<0.001** |
| B437 | *Soilibacillus* sp. | 0.324 ± 0.005ᶜ | 0.227 ± 0.008ᵈ | 0.742 ± 0.026ᵇ | 0.898 ± 0.002ᵃ | **<0.001** |
| B451 | *Pseudomonas* sp. | 0.181 ± 0.021ᵇ | 0.747 ± 0.040ᵃ | 0.735 ± 0.030ᵃ | 0.736 ± 0.028ᵃ | **<0.001** |
| B487 | *Bacillus licheniformis* | 0.236 ± 0.001ᵇ | 0.837 ± 0.030ᵃ | 0.853 ± 0.097ᵃ | 0.898 ± 0.058ᵃ | **<0.001** |
| B527 | *Paenibacillus amylolyticus* | 0.240 ± 0.014ᶜ | 0.381 ± 0.056ᵇ | 0.898 ± 0.021ᵃ | 0.879 ± 0.084ᵃ | **<0.001** |
| B573 | *Pseudomonas asgharzadehiana* | 0.188 ± 0.014ᶜ | 0.886 ± 0.020ᵃ | 0.814 ± 0.054ᵃᵇ | 0.798 ± 0.020ᵇ | **<0.001** |
| B588 | *Pseudomonas simiae* | 0.703 ± 0.035ᶜ | 0.811 ± 0.009ᵃ | 0.757 ± 0.010ᵇ | 0.765 ± 0.005ᵇ | **<0.001** |
| B598 | *Paenibacillus amylolyticus* | 0.625 ± 0.062ᵃᵇ | 0.530 ± 0.118ᵇ | 0.869 ± 0.171ᵃ | 0.634 ± 0.115ᵃᵇ | **0.048** |
| B602 | *Paenibacillus amylolyticus* | 0.507 ± 0.026ᵇ | 0.585 ± 0.054ᵇ | 0.504 ± 0.104ᵇ | 0.774 ± 0.046ᵃ | **0.003** |
| B604 | *Bacillus licheniformis* | 0.406 ± 0.004ᶜ | 0.570 ± 0.002ᵇ | 0.699 ± 0.082ᵃ | 0.706 ± 0.040ᵃ | **<0.001** |
| B641 | *Paenibacillus nuruki* | 0.365 ± 0.002ᶜ | 0.442 ± 0.013ᵇ | 0.554 ± 0.043ᵃ | 0.463 ± 0.036ᵇ | **<0.001** |

**Table S7**. Salinity tolerance of *Pseudomonadaceae*. *Bacillaceae* and *Paenibacillaceae* at different NaCl concentrations. Data are presented as means of absorbance (OD_600_) ± standard deviation (n = 3). Different letters indicate significant differences among NaCl concentrations within the same isolate. according to Tukey’s test at 5% significance.

| Strain | Species | Salt concentration | | | |  |
| --- | --- | --- | --- | --- | --- | --- |
|  |  | **NaCl 0%** | **NaCl 2.5%** | **NaCl 5%** | **NaCl 10%** | ***Fprob*** |
| B29 | *Psychrobacillus psychrodurans* | 0.315 ± 0.009ᵃ | 0.279 ± 0.004ᵇ | 0.201 ± 0.004ᶜ | 0.201 ± 0.004ᵈ | **<0.001** |
| B41 | *Pseudomonas* sp. | 0.865 ± 0.023ᵃ | 0.883 ± 0.023ᵃ | 0.615 ± 0.034ᵇ | 0.615 ± 0.034ᶜ | **<0.001** |
| B76 | *Paenibacillus amylolyticus* | 0.186 ± 0.006ᵇ | 0.214 ± 0.003ᵃ | 0.097 ± 0.012ᶜ | 0.097 ± 0.012ᶜ | **<0.001** |
| B93 | *Paenibacillus nuruki* | 0.207 ± 0.009ᵃ | 0.209 ± 0.014ᵃ | 0.045 ± 0.003ᵇ | 0.045 ± 0.003ᵇ | **<0.001** |
| B110 | *Pseudomonas simiae* | 0.809 ± 0.077ᵃ | 0.822 ± 0.020ᵃ | 0.693 ± 0.047ᵃ | 0.693 ± 0.047ᵇ | **<0.001** |
| B126 | *Paenibacillus amylolyticus* | 0.116 ± 0.004ᵇ | 0.187 ± 0.007ᵃ | 0.059 ± 0.001ᶜ | 0.059 ± 0.001ᵈ | **<0.001** |
| B192 | *Terribacillus saccharophilus* | 0.177 ± 0.162ᵃ | 0.110 ± 0.002ᵃ | 0.110 ± 0.002ᵃ | 0.110 ± 0.002ᵃ | 0.407 |
| B194 | *Paenibacillus amylolyticus* | 0.202 ± 0.034ᵃ | 0.102 ± 0.003ᵇ | 0.050 ± 0.003ᶜ | 0.050 ± 0.003ᶜ | **<0.001** |
| B198 | *Peribacillus frigorotolerans* | 0.032 ± 0.003ᵈ | 0.058 ± 0.001ᵇ | 0.142 ± 0.004ᵃ | 0.142 ± 0.004ᶜ | **<0.001** |
| B201 | *Paenibacillus nuruki* | 0.306 ± 0.003ᵃ | 0.143 ± 0.026ᵇ | 0.016 ± 0.000ᶜ | 0.016 ± 0.000ᶜ | **<0.001** |
| B216 | *Peribacillus frigorotolerans* | 0.276 ± 0.007ᵃ | 0.198 ± 0.013ᵇ | 0.262 ± 0.007ᵃ | 0.262 ± 0.007ᶜ | **<0.001** |
| B221 | *Bacillus pumilus* | 0.211 ± 0.008ᶜ | 0.342 ± 0.057ᵇ | 0.590 ± 0.013ᵃ | 0.590 ± 0.013ᶜ | **<0.001** |
| B254 | *Pseudomonas tritici* | 0.578 ± 0.014ᵇ | 0.704 ± 0.026ᵃ | 0.299 ± 0.031ᶜ | 0.299 ± 0.031ᵈ | **<0.001** |
| B287 | *Paenibacillus amylolyticus* | 0.274 ± 0.007ᵃ | 0.215 ± 0.010ᵇ | 0.184 ± 0.004ᶜ | 0.184 ± 0.004ᵈ | **<0.001** |
| B293 | *Bacillus velezensis* | 0.165 ± 0.003ᶜ | 0.292 ± 0.006ᵇ | 0.435 ± 0.031ᵃ | 0.435 ± 0.031ᵈ | **<0.001** |
| B338 | *Paenibacillus amylolyticus* | 0.225 ± 0.003ᵃ | 0.177 ± 0.007ᶜ | 0.205 ± 0.007ᵇ | 0.205 ± 0.007ᵃ | **<0.001** |
| B342 | *Paenibacillus pabuli* | 0.135 ± 0.006ᶜ | 0.286 ± 0.037ᵃ | 0.091 ± 0.002ᶜ | 0.091 ± 0.002ᵇ | **<0.001** |
| B353 | *Bacillus velezensis* | 0.328 ± 0.191ᵃ | 0.138 ± 0.010ᵃ | 0.382 ± 0.007ᵃ | 0.382 ± 0.007ᵃ | 0.060 |
| B362 | *Pseudomonas iranensis* | 0.744 ± 0.090ᵇ | 0.948 ± 0.012ᵃ | 0.612 ± 0.040ᶜ | 0.612 ± 0.040ᵈ | **<0.001** |
| B367 | *Pseudomonas flourescens* | 0.712 ± 0.037ᵇ | 0.825 ± 0.019ᵃ | 0.784 ± 0.030ᵃ | 0.784 ± 0.030ᶜ | **<0.001** |
| B393 | *Bacillus pumilus* | 0.330 ± 0.008ᵇ | 0.169 ± 0.003ᶜ | 0.481 ± 0.004ᵃ | 0.481 ± 0.004ᶜ | **<0.001** |
| B411 | *Pseudomonas* sp. | 0.798 ± 0.005ᵇ | 0.845 ± 0.002ᵃ | 0.657 ± 0.014ᶜ | 0.657 ± 0.014ᵈ | **<0.001** |
| B437 | *Soilibacillus* sp. | 0.198 ± 0.004ᵇ | 0.146 ± 0.003ᶜ | 0.035 ± 0.001ᵈ | 0.035 ± 0.001ᵃ | **<0.001** |
| B451 | *Pseudomonas* sp. | 0.610 ± 0.003ᵇ | 0.722 ± 0.037ᵃ | 0.694 ± 0.022ᵃ | 0.694 ± 0.022ᶜ | **<0.001** |
| B487 | *Bacillus licheniformis* | 0.253 ± 0.006ᵃ | 0.273 ± 0.021ᵃ | 0.035 ± 0.001ᵇ | 0.035 ± 0.001ᵇ | **<0.001** |
| B527 | *Paenibacillus amylolyticus* | 0.149 ± 0.003ᵇ | 0.137 ± 0.004ᵇ | 0.403 ± 0.004ᵃ | 0.403 ± 0.004ᶜ | **<0.001** |
| B573 | *Pseudomonas asgharzadehiana* | 0.567 ± 0.016ᶜ | 0.842 ± 0.013ᵃ | 0.664 ± 0.050ᵇ | 0.664 ± 0.050ᵈ | **<0.001** |
| B588 | *Pseudomonas simiae* | 0.780 ± 0.009ᵇ | 0.853 ± 0.033ᵃ | 0.798 ± 0.008ᵇ | 0.798 ± 0.008ᶜ | **<0.001** |
| B598 | *Paenibacillus amylolyticus* | 0.185 ± 0.004ᵃ | 0.064 ± 0.006ᵇ | 0.024 ± 0.000ᶜ | 0.024 ± 0.000ᶜ | **<0.001** |
| B602 | *Paenibacillus amylolyticus* | 0.229 ± 0.000ᵃ | 0.181 ± 0.005ᵃ | 0.218 ± 0.007ᵃ | 0.218 ± 0.007ᵇ | **0.006** |
| B604 | *Bacillus licheniformis* | 0.217 ± 0.014ᵃ | 0.147 ± 0.018ᵇ | 0.131 ± 0.003ᵇ | 0.131 ± 0.003ᵇ | **<0.001** |
| B641 | *Paenibacillus nuruki* | 0.225 ± 0.009ᵇ | 0.191 ± 0.006ᶜ | 0.394 ± 0.007ᵃ | 0.394 ± 0.007ᵈ | **<0.001** |

**Table S8**. Water stress tolerance of *Pseudomonadaceae*. *Bacillaceae* and *Paenibacillaceae* under different concentrations of PEG 4000. Data are presented as means of absorbance (OD_600_) ± standard deviation (n = 3). Different letters indicate significant differences among PEG concentrations within the same isolate. according to Tukey’s test at 5% significance.

| Strain | Species | PEG 4000 Concentration | | | | |  |
| --- | --- | --- | --- | --- | --- | --- | --- |
|  |  | **PEG 0%** | **PEG 5%** | **PEG 10%** | **PEG 20%** | **PEG 30%** | ***Fprob*** |
| B29 | *Psychrobacillus psychrodurans* | 0.006 ± 0.000ᵇ | 0.026 ± 0.010ᵃᵇ | 0.036 ± 0.004ᵃ | 0.045 ± 0.001ᵃ | 0.029 ± 0.001ᵃ | **<0.001** |
| B41 | *Pseudomonas* sp. | 0.786 ± 0.002ᵃ | 0.631 ± 0.000ᵇ | 0.485 ± 0.002ᶜ | 0.257 ± 0.004ᵈ | 0.009 ± 0.004ᵉ | **<0.001** |
| B76 | *Paenibacillus amylolyticus* | 0.182 ± 0.003ᵃ | 0.154 ± 0.031ᵃᵇ | 0.115 ± 0.033ᵇ | 0.052 ± 0.002ᶜ | 0.028 ± 0.003ᶜ | **<0.001** |
| B93 | *Paenibacillus nuruki* | 0.197 ± 0.006ᵃ | 0.152 ± 0.008ᵇ | 0.138 ± 0.028ᵇ | 0.053 ± 0.004ᶜ | 0.012 ± 0.002ᶜ | **0.006** |
| B110 | *Pseudomonas simiae* | 0.846 ± 0.019ᵃ | 0.702 ± 0.008ᵇ | 0.595 ± 0.026ᶜ | 0.323 ± 0.018ᵈ | 0.008 ± 0.003ᵉ | **<0.001** |
| B126 | *Paenibacillus amylolyticus* | 0.180 ± 0.003ᵃ | 0.120 ± 0.002ᵇ | 0.076 ± 0.004ᶜ | 0.050 ± 0.006ᵈ | 0.044 ± 0.007ᵈ | **<0.001** |
| B192 | *Terribacillus saccharophilus* | 0.472 ± 0.006ᵃ | 0.083 ± 0.003ᵇ | 0.046 ± 0.013ᶜ | 0.017 ± 0.005ᵈ | 0.006 ± 0.005ᵈ | **<0.001** |
| B194 | *Paenibacillus amylolyticus* | 0.393 ± 0.006ᵃ | 0.149 ± 0.002ᵇ | 0.081 ± 0.004ᶜ | 0.054 ± 0.004ᵈ | 0.026 ± 0.008ᵉ | **<0.001** |
| B198 | *Peribacillus frigorotolerans* | 0.363 ± 0.004ᵃ | 0.090 ± 0.007ᵇ | 0.063 ± 0.006ᶜ | 0.043 ± 0.004ᶜ | 0.010 ± 0.012ᵈ | **<0.001** |
| B201 | *Paenibacillus nuruki* | 0.240 ± 0.006ᵃ | 0.200 ± 0.006ᵇ | 0.126 ± 0.008ᶜ | 0.073 ± 0.002ᵈ | 0.039 ± 0.004ᵉ | **0.006** |
| B216 | *Peribacillus frigorotolerans* | 0.252 ± 0.003ᵃ | 0.142 ± 0.004ᵇ | 0.090 ± 0.003ᶜ | 0.065 ± 0.004ᵈ | 0.028 ± 0.006ᵉ | **<0.001** |
| B221 | *Bacillus pumilus* | 0.403 ± 0.006ᵃ | 0.173 ± 0.002ᵇ | 0.129 ± 0.003ᶜ | 0.075 ± 0.003ᵈ | 0.032 ± 0.014ᵉ | **<0.001** |
| B254 | *Pseudomonas tritici* | 0.952 ± 0.035ᵃ | 0.677 ± 0.016ᵇ | 0.580 ± 0.020ᶜ | 0.203 ± 0.002ᵈ | 0.028 ± 0.012ᵉ | **<0.001** |
| B287 | *Paenibacillus amylolyticus* | 0.189 ± 0.002ᵃ | 0.131 ± 0.009ᵇ | 0.089 ± 0.001ᶜ | 0.051 ± 0.003ᵈ | 0.036 ± 0.003ᵈ | **<0.001** |
| B293 | *Bacillus velezensis* | 0.216 ± 0.009ᵃ | 0.068 ± 0.011ᵇ | 0.100 ± 0.014ᶜ | 0.036 ± 0.003ᵈ | 0.005 ± 0.008ᵉ | **<0.001** |
| B338 | *Paenibacillus amylolyticus* | 0.180 ± 0.014ᵃ | 0.142 ± 0.001ᵇ | 0.087 ± 0.003ᶜ | 0.050 ± 0.010ᵈ | 0.033 ± 0.001ᵈ | **0.006** |
| B342 | *Paenibacillus pabuli* | 0.237 ± 0.002ᵃ | 0.138 ± 0.008ᵇ | 0.091 ± 0.003ᶜ | 0.050 ± 0.002ᵈ | 0.039 ± 0.009ᵈ | **<0.001** |
| B353 | *Bacillus velezensis* | 0.286 ± 0.001ᵃ | 0.088 ± 0.007ᵇ | 0.058 ± 0.012ᶜ | 0.042 ± 0.008ᶜ | 0.005 ± 0.006ᵈ | **<0.001** |
| B362 | *Pseudomonas iranensis* | 0.783 ± 0.003ᵃ | 0.612 ± 0.031ᵇ | 0.497 ± 0.002ᶜ | 0.245 ± 0.002ᵈ | 0.029 ± 0.001ᵉ | **<0.001** |
| B367 | *Pseudomonas flourescens* | 0.773 ± 0.067ᵃ | 0.659 ± 0.005ᵇ | 0.549 ± 0.011ᶜ | 0.301 ± 0.011ᵈ | 0.000 ± 0.001ᵉ | **<0.001** |
| B393 | *Bacillus pumilus* | 0.451 ± 0.005ᵃ | 0.159 ± 0.028ᵇ | 0.128 ± 0.001ᵇ | 0.074 ± 0.000ᶜ | 0.033 ± 0.003ᵈ | **<0.001** |
| B411 | *Pseudomonas* sp. | 0.832 ± 0.028ᵃ | 0.760 ± 0.080ᵃ | 0.529 ± 0.012ᵇ | 0.252 ± 0.002ᶜ | 0.001 ± 0.000ᵈ | **0.006** |
| B437 | *Solibacillus* sp. | 0.244 ± 0.016ᵃ | 0.147 ± 0.005ᵇ | 0.108 ± 0.007ᶜ | 0.059 ± 0.001ᵈ | 0.026 ± 0.003ᵉ | **<0.001** |
| B451 | *Pseudomonas* sp. | 0.769 ± 0.009ᵃ | 0.526 ± 0.001ᵇ | 0.463 ± 0.016ᶜ | 0.156 ± 0.009ᵈ | 0.015 ± 0.003ᵉ | **<0.001** |
| B487 | *Bacillus licheniformis* | 0.326 ± 0.002ᵃ | 0.063 ± 0.002ᵇ | 0.080 ± 0.003ᶜ | 0.044 ± 0.010ᵈ | 0.012 ± 0.005ᵉ | **<0.001** |
| B527 | *Paenibacillus amylolyticus* | 0.040 ± 0.006ᵇ | 0.102 ± 0.020ᵃ | 0.092 ± 0.003ᵃ | 0.031 ± 0.001ᵇ | 0.022 ± 0.003ᵇ | **<0.001** |
| B573 | *Pseudomonas asgharzadehiana* | 0.868 ± 0.015ᵃ | 0.663 ± 0.014ᵇ | 0.521 ± 0.033ᶜ | 0.211 ± 0.005ᵈ | 0.008 ± 0.001ᵉ | **<0.001** |
| B588 | *Pseudomonas simiae* | 0.822 ± 0.006ᵃ | 0.643 ± 0.031ᵇ | 0.513 ± 0.004ᶜ | 0.247 ± 0.007ᵈ | 0.016 ± 0.002ᵉ | **0.006** |
| B598 | *Paenibacillus amylolyticus* | 0.051 ± 0.002ᶜ | 0.149 ± 0.023ᵃ | 0.091 ± 0.003ᵇ | 0.061 ± 0.005ᵇᶜ | 0.037 ± 0.006ᶜ | **<0.001** |
| B602 | *Paenibacillus amylolyticus* | 0.193 ± 0.004ᵃ | 0.150 ± 0.008ᵇ | 0.089 ± 0.009ᶜ | 0.035 ± 0.005ᵈ | 0.035 ± 0.001ᵈ | **<0.001** |
| B604 | *Bacillus licheniformis* | 0.324 ± 0.001ᵃ | 0.062 ± 0.002ᵇ | 0.080 ± 0.004ᶜ | 0.044 ± 0.010ᵈ | 0.012 ± 0.005ᵉ | **<0.001** |
| B641 | *Paenibacillus nuruki* | 0.544 ± 0.012ᵃ | 0.152 ± 0.011ᵇ | 0.103 ± 0.009ᶜ | 0.049 ± 0.002ᵈ | 0.017 ± 0.003ᵉ | **<0.001** |

**Figure S1.** Cont.

**Figure S1.** Phylogenetic relationships generated from maximum likelihood analysis based on 16S rRNA. *gyrB* and *rpoD* sequence data from *the family Pseudomonadaceae*. The ML tree is rooted to *Azotobacter* *salinestris* KACC 13899 and was built using the general time-reversible (GTR) model assuming a gamma distribution and invariant sites. Maximum likelihood (ML) bootstrap values greater than 70% are shown at the nodes. The ex-type strains are shown in bold. The newly generated sequences are shown in orange. The scale bar indicates the evolutionary distance.

**Figure S2**. Phylogenetic relationships generated from maximum likelihood analysis based on 16S rRNA and *gyrB* sequence data from members of the family *Bacillaceae* and *Paenibacillaceae*. The ML tree was built using the Kimura 2 parameter model assuming a gamma distribution and invariant sites. Maximum likelihood (ML) bootstrap values greater than 70% are shown at the nodes. The ex-type strains are shown in bold. The newly generated sequences are shown in orange. The scale bar indicates the evolutionary distance.

**Figure S3.** Heatmap showing the performance of *Pseudomonadaceae* regarding indole-3-acetic acid (IAA) production. phosphate solubilization index (PSI). and nitrogen fixation (NF) ability. Data are presented as means (n=3) and normalized using Min–Max Scaling (linear normalization to the range [0–1]).
